# Genotype-by-environment interactions for reproduction, body composition, and growth traits in maternal-line pigs based on single-step genomic reaction norms

**DOI:** 10.1186/s12711-021-00645-y

**Published:** 2021-06-17

**Authors:** Shi-Yi Chen, Pedro H. F. Freitas, Hinayah R. Oliveira, Sirlene F. Lázaro, Yi Jian Huang, Jeremy T. Howard, Youping Gu, Allan P. Schinckel, Luiz F. Brito

**Affiliations:** 1 Department of Animal Sciences, Purdue University, West Lafayette, IN 47907 USA; 2 Farm Animal Genetic Resources Exploration and Innovation Key Laboratory of Sichuan Province, Sichuan Agricultural University, Chengdu, 611130 Sichuan China; 3 Centre for Genetic Improvement of Livestock, Department of Animal Biosciences, University of Guelph, Guelph, ON N1G 2W1 Canada; 4 Department of Animal Science, College of Agricultural and Veterinary Sciences, São Paulo State University (UNESP), Jaboticabal, SP 14884-900 Brazil; 5 Smithfield Premium Genetics, Rose Hill, NC USA

## Abstract

**Background:**

There is an increasing need to account for genotype-by-environment (G × E) interactions in livestock breeding programs to improve productivity and animal welfare across environmental and management conditions. This is even more relevant for pigs because selection occurs in high-health nucleus farms, while commercial pigs are raised in more challenging environments. In this study, we used single-step homoscedastic and heteroscedastic genomic reaction norm models (RNM) to evaluate G × E interactions in Large White pigs, including 8686 genotyped animals, for reproduction (total number of piglets born, TNB; total number of piglets born alive, NBA; total number of piglets weaned, NW), growth (weaning weight, WW; off-test weight, OW), and body composition (ultrasound muscle depth, MD; ultrasound backfat thickness, BF) traits. Genetic parameter estimation and single-step genome-wide association studies (ssGWAS) were performed for each trait.

**Results:**

The average performance of contemporary groups (CG) was estimated and used as environmental gradient in the reaction norm analyses. We found that the need to consider heterogeneous residual variance in RNM models was trait dependent. Based on estimates of variance components of the RNM slope and of genetic correlations across environmental gradients, G × E interactions clearly existed for TNB and NBA, existed for WW but were of smaller magnitude, and were not detected for NW, OW, MD, and BF. Based on estimates of the genetic variance explained by the markers in sliding genomic windows in ssGWAS, several genomic regions were associated with the RNM slope for TNB, NBA, and WW, indicating specific biological mechanisms underlying environmental sensitivity, and dozens of novel candidate genes were identified. Our results also provided strong evidence that the X chromosome contributed to the intercept and slope of RNM for litter size traits in pigs.

**Conclusions:**

We provide a comprehensive description of G × E interactions in Large White pigs for economically-relevant traits and identified important genomic regions and candidate genes associated with GxE interactions on several autosomes and the X chromosome. Implementation of these findings will contribute to more accurate genomic estimates of breeding values by considering G × E interactions, in order to genetically improve the environmental robustness of maternal-line pigs.

**Supplementary Information:**

The online version contains supplementary material available at 10.1186/s12711-021-00645-y.

## Background

In recent years, increased attention has been directed towards the genetic evaluation of genotype-by-environment (G × E) interactions for economically important traits in livestock [1–3]. In pigs, dissecting G × E interactions is even more important because breeding programs are usually conducted independently in nucleus farms, which differ considerably from commercial farms in terms of environmental conditions (e.g., climate, health status, nutrition, and management practices). Such heterogeneous environments can decrease the accuracy of estimated breeding values when G × E interactions are not accounted for in the genetic evaluation models [4]. Furthermore, the magnitude or ranking of estimated breeding values of selection candidates could differ between environments due to G × E interactions, which means that the animals that are selected based on their estimated breeding values for a certain environment might not perform well under divergent environmental conditions. Selective breeding of animals that perform well across environments is expected to improve productivity, health, and welfare of the animals and, therefore, the profitability of the swine industry [5–8].

From a technological point of view, the evaluation of G × E interactions has been facilitated by recent advances in genomic technologies and analytical methods [9, 10], because it no longer requires the recording of phenotypes on close relatives in multiple environments (e.g. geographically distributed paternal half-sib offspring [11]). To date, only a few studies on genomic evaluation accounting for G × E interactions have been published for maternal-line pigs [12–14]. The selection indexes for terminal sire and maternal line pigs are different, and therefore, the biological mechanisms that underlie heat tolerance could also differ (e.g., greater metabolic heat production in breeds selected for higher milk production, i.e., larger litters).

Analysis of G × E interactions can be accomplished by two main approaches [2]. The first approach considers that phenotypic measurements obtained in different environments are different but genetically-correlated traits, with analyses using multiple-trait methods. This approach is preferentially applied for categorical environmental descriptors, such as temperate versus tropical climates [15], or organic versus conventional production systems [16]. In the second approach, G × E interactions are directly modeled using a reaction norm model (RNM), which is recommended for continuous environmental descriptors, such as the temperature-humidity index (THI) [12, 13] or the estimated average performance of contemporary groups (CG) [14, 17]. In RNM, the phenotypic values of each animal are regressed on the environmental variable to estimate breeding values for the regression intercept and slope for each animal [18, 19]. The RNM approach can also be used to model nonlinear G × E interactions [20]. The RNM has been used for the genomic evaluation of G × E interactions in pigs [12–14, 21] and in dairy and beef cattle [20, 22, 23].

In the swine industry, environmental variation can be described in terms of quantitative differences in environmental THI, nutrition, management practices, health status, and other unknown factors, noting that accurately quantifying environmental variation regarding nutrition and management practices is not easy, because these cannot be summarized in an index such as THI [12]. An alternative and commonly applied method is to use an estimate of the average performance of CG as a proxy for overall differences in environmental conditions [13].

In early studies of G × E interactions in livestock, individuals recorded in different environments were usually connected based on breed origin, sire progeny groups, or pedigree records [2]. In the genomics era, the single-step genomic best linear unbiased prediction method (ssGBLUP) is typically used for genomic evaluation because it can combine pedigree and genotype information in a single analysis [24, 25]. As a result, combining the ssGBLUP method with RNM (i.e., single-step genomic RNM) is also becoming popular in the evaluation of G × E interactions in livestock [12–14]. Within the ssGBLUP framework, the single-step genome-wide association study (ssGWAS) approach has been successfully used to include phenotypic information from non-genotyped individuals [26]. Thus, inclusion of single nucleotide polymorphism (SNP) genotypes in the evaluation of G × E interactions enables the detection of SNPs that are associated with environment-robust or environment-sensitive characteristics [20, 23].

Due to its specific gene content and dosage regulation, the X chromosome can have substantial effects on the reproductive performance of both female and male mammals [27]. However, SNPs located on the X chromosome tend to be ignored in genomic analyses of complex traits, mainly because of the analytical challenges and biological considerations [28–30]. Recently, the inclusion of the X chromosome in such analyses was suggested to improve the accuracy of genomic prediction in both dairy and beef cattle [28, 31]. To the best of our knowledge, SNPs located on the X chromosome have rarely been included in genomic analyses of the pig, especially of G × E interactions based on reproduction traits, and, thus, limited information is available on genomic polymorphisms and functional genes on the X chromosome that are associated with reproduction traits [13, 14, 32]. Therefore, our main objectives were to: (1) provide a comprehensive description of G × E interaction effects for various reproduction, growth, and body composition traits in Large White pigs, including SNPs on the X chromosome; (2) dissect the genomic regions that have effects on the RNM slope for G × E interaction effects; and (3) reveal candidate genes involved in the biological mechanisms that underlie G × E interactions in maternal-line pigs.

## Methods

### Trait definition and data editing

All the datasets analyzed in this study were provided by Smithfield Premium Genetics (Rose Hill, NC, USA). Phenotypic records were collected on Large White pigs born from January 2004 to December 2019 on 33 farms that were geographically distributed across North America. Seven traits were analyzed [see Additional file 1: Table S1], including three reproduction traits (total number of piglets born, TNB; total number of piglets born alive, NBA; and total number of piglets weaned, NW), two growth traits (weaning weight, WW; and off-test weight, OW), and two body composition traits (ultrasound muscle depth, MD; and ultrasound backfat thickness, BF). Off-test weight was defined as the body weight recorded at the end of the test period, when the animals were on average (±SD, standard deviation) 151 ± 17 days old. CG were defined by concatenating farrowing year, season, and farm for the reproduction traits, and birth year, season, and farm for the growth and body composition traits. CG with less than 10 records were removed from the analyses. Outliers were discarded if they deviated by more than 3.5 SD from the trait mean. Descriptive statistics of the phenotypic data and CG are in Table 1.

**Table 1:** Descriptive statistics for trait phenotypes and effects included in the mixed model for each trait

Trait	Descriptive statistics			Effects included in the mixed models		
	Number of records	SD	Number (estimates) of CG	Fixed effects	Covariates	Random effects
TNB	186,189	3.41	474 (− 2.90–3.50)	FP, CG_R	fAge, fAge2	*a*, *pe*, *ce*
NBA	185,824	3.22	474 (− 3.04–3.26)	FP, CG_R	fAge, fAge2	*a*, *pe*, *ce*
NW	8164	2.74	140 (− 3.69–2.70)	FP, CG_R	fAge, wAge, wAge2	*a*, *pe*, *ce*
WW	27,412	1.87	75 (− 1.49–2.80)	Sex, BP, CG_G	wAge	*a*, *ce*
OW	101,541	25.63	256 (− 1.93–2.01)	Sex, BP, CG_G	oAge, oAge2, wAge, wAge2	*a*, *ce*
MD	20,149	6.45	86 (− 1.92–2.84)	Sex, BP, CG_G	oAge, oAge2, wAge, wAge2	*a*, *ce*
BF	20,175	4.13	87 (− 2.72–2.46)	Sex, BP, CG_G	oAge, oAge2, wAge	*a*, *ce*

TNB, total number of piglets born; NBA, number of piglets born alive; NW, number of piglets weaned; WW, weaning weight (kg); OW, off-test weight (kg); MD, ultrasound muscle depth (mm); BF, ultrasound backfat thickness (mm)

Number of records, number of phenotypic records after quality control; SD: standard deviation; CG, contemporary group; the effects of CG are the standardized ranges

FP, farrowing parity; BP, birth parity; CG_R, reproduction contemporary group; CG_G, growth contemporary group

fAge, linear effect of farrowing age; fAge2, quadratic effect of farrowing age; wAge, linear effect of weaning age; wAge2, quadratic effect of weaning age; oAge, linear effect of off-test age; oAge2, quadratic effect of off-test age

*a*, additive genetic effect; *pe,* animal permanent environmental effect across parities; *ce,* litter effect

### Genomic datasets

After phenotypic quality control, the raw pedigree file included 265,943 animals across more than 10 generations. Ten generations were traced back when calculating the genetic and genomic relationships. In total, 8992 animals were initially genotyped using the PorcineSNP10K (8652 SNPs for 886 animals), PorcineSNP50K (50,549 SNPs for 5706 animals), PorcineSNP60K (57,019 SNPs for 865 animals), or PorcineSNP80K (64,577 SNPs for 1535 animals) Bead Chips (Illumina, San Diego, CA, USA). Animals with genotyping call rates lower than 90% were discarded (N = 224 animals). For animals that were genotyped with more than one SNP panel (N = 82), the data from the higher-density SNP panel were kept for further analysis. Finally, 8686 animals (7017 females and 1669 males) with genotype information remained in the dataset.

We imputed the genotypes from low- to high-density SNP panels using the FImpute software with default parameters [33] based on the following two steps: (1) imputation from the 10K panel (6111 SNPs) to the 50K panel (49,944 SNPs); and (2) imputation from the 50K or 60K (39,567 SNPs) panels to the 80K panel (64,577 SNPs). Prior to imputation, SNPs that were exclusively included in the lower density panels were removed, including 2541 SNPs in the PorcineSNP10K, 605 SNPs in the imputed PorcineSNP50K, and 26,139 SNPs in the PorcineSNP60K panels. The accuracy of genotype imputation was not investigated here, but high imputation accuracies were obtained for the same breed and based on a smaller reference population, even when imputing from much lower SNP panel densities [34]. After imputation, the SNP data was subjected to quality control using the BLUPF90 programs during the genomic analyses [35, 36] by requiring a call rate higher than 0.90, a minor allele frequency higher than 0.01, and a difference between observed and expected heterozygote frequencies smaller than 0.15. In the end, 55,375 informative SNPs on 18 autosomes (N = 53,031, 95.8%) and the X chromosome (N = 2344, 4.2%) for 8686 animals were included in subsequent analyses.

### Model development, environmental descriptors, and genetic analyses

All recorded categorical fixed effects (i.e., sex, parity, and CG) and covariates (linear and quadratic effects for farrowing age, weaning age, and off-test age) were selected for inclusion in the model based on the backward elimination procedure (*P* < 0.05) of the *lm* function in the R software [37], separately for each trait. Three random effects were subjected to model comparisons on the basis of the Akaike Information Criterion (AIC) values, using the AIREMLF90 software [35, 36]: an animal (additive genetic) effect ($a$), a permanent environmental effect across parities ($pe$), and a litter effect ($ce$). The final model for each trait is given in Table 1 and Additional file 1: Table S2.

Similar to recent studies in dairy and beef cattle [20, 23], the average performance (or effect) of CG was estimated by ssGBLUP and used as environmental descriptors. Thus, for each trait, the effect of each CG was estimated using a linear model containing all the systematic effects described above and the best linear unbiased estimator (BLUE) method. Estimates of CG were standardized to have a zero mean and a SD equal to 1 for each trait, and CG that deviated by more than 3.5 SD from the mean were removed. The standardized estimates of CG were used as environmental gradients for the genetic evaluation of G × E interactions, following previous studies [14, 20, 23, 38].

Genetic analyses were performed using ssGBLUP and RNM, the fixed and random effects listed in Table 1, and under homogenous and heterogeneous residual variances. The first RNM (RNM1), which considered a homogenous residual variance, was defined as:

$$y_{{ij}} = {\mathbf{x}}_{j}^{'} {\varvec{\upbeta}} + b\hat{\theta }_{i} + \sum (n_{{0_{j} }} + n_{{1_{j} }} \hat{\theta }_{i} ) + ~e_{{ij}} ,$$

where $y_{{ij}}$ is the phenotypic observation of animal $j$ in CG $i$; ${\varvec{\upbeta}}$ is the vector of fixed effects/covariates described in Table 1, together with its row incidence vector ${\mathbf{x}}_{j}^{'}$; $\hat{\theta }_{i}$ is the estimated effect of CG $i$ from the previous step, $b$ is the overall fixed regression coefficient of $y_{{ij}}$ on $\hat{\theta }_{i}$; $n_{{0_{j} }}$ and $n_{{1_{j} }}$ are the RNM intercept and slope of animal $j$ regressed on $\hat{\theta }_{i}$ for random effect $n$ ($n \in \left\{ {a,pe,ce} \right\}$, as described in Table 1 for each trait); and $e_{{ij}}$ is the random residual of animal $j$ in CG $i$. The assumed covariance structures were as follows:

$$\left[ {\begin{array}{*{20}c} {{\mathbf{a}}_{{\mathbf{0}}} } \\ {{\mathbf{a}}_{{\mathbf{1}}} } \\ \end{array} } \right]\sim N\left( {{\mathbf{0}},{\text{~}}{\mathbf{H}} \otimes \left[ {\begin{array}{*{20}c} {\sigma _{{a_{0} }}^{2} } & {\sigma _{{a_{0} a_{1} }} } \\ {\sigma _{{a_{0} a_{1} }} } & {\sigma _{{a_{1} }}^{2} } \\ \end{array} } \right]} \right),$$

and

$$\left[ {\begin{array}{*{20}c} {\begin{array}{*{20}c} {{\mathbf{pe}}_{{\mathbf{0}}} } \\ {{\mathbf{pe}}_{{\mathbf{1}}} } \\ {{\mathbf{ce}}_{{\mathbf{0}}} } \\ \end{array} } \\ {{\mathbf{ce}}_{{\mathbf{1}}} } \\ {\mathbf{e}} \\ \end{array} } \right]\sim N\left( {{\mathbf{0}},~{\mathbf{I}} \otimes \left[ {\begin{array}{*{20}c} {\sigma _{{pe_{0} }}^{2} } & {\sigma _{{pe_{0} pe_{1} }} } & 0 & 0 & 0 \\ {\sigma _{{pe_{0} pe_{1} }} } & {\sigma _{{pe_{1} }}^{2} } & 0 & 0 & 0 \\ 0 & 0 & {\sigma _{{ce_{1} }}^{2} } & {\sigma _{{ce_{0} ce_{1} }} } & 0 \\ 0 & 0 & {\sigma _{{ce_{0} ce_{1} }} } & {\sigma _{{ce_{1} }}^{2} } & 0 \\ 0 & 0 & 0 & 0 & {\sigma _{e}^{2} } \\ \end{array} } \right]} \right),$$

where $\sigma _{{n_{0} }}^{2}$, $\sigma _{{n_{1} }}^{2}$ and $\sigma _{{n_{0} n_{1} }}$ are the variance of coefficient $n_{{0_{j} }}$, the variance of coefficient $n_{{1_{j} }}$, and the covariance between $n_{{0_{j} }}$ and $n_{{1_{j} }}$, respectively. ${\mathbf{a}}$, ${\mathbf{pe}}$, and ${\mathbf{ce}}$ are the vectors of the direct genetic, permanent environment, and common environment effects, respectively, for the intercept (“0”) and slope (“1”) terms. ${\mathbf{H}}$ is the hybrid relationship matrix that combines pedigree and genomic relationships [39], and ${\mathbf{I}}$ is an identity matrix. The inverse of ${\mathbf{H}}$ (${\mathbf{H}}^{{ - 1}} )$ was computed as [24]:

$${\mathbf{H}}^{{{\mathbf{ - 1}}}} = {\mathbf{A}}^{{{\mathbf{ - 1}}}} \left[ {\begin{array}{*{20}c} {\mathbf{0}} & {\mathbf{0}} \\ {\mathbf{0}} & {{\mathbf{G}}^{{{\mathbf{ - 1}}}} {\mathbf{ - A}}_{{{\mathbf{22}}}}^{{{\mathbf{ - 1}}}} } \\ \end{array} } \right],$$

where ${\mathbf{A}}^{{ - 1}}$ is the inverse of the numerator relationship matrix ${\mathbf{A}}$, ${\mathbf{A}}_{{22}}^{{ - 1}}$ is the inverse of ${\mathbf{A}}$ for the genotyped animals, and ${\mathbf{G}}^{{ - 1}}$ is the inverse of the genomic-based relationship matrix ${\mathbf{G}}$.

The second RNM (RNM2) was similar to RNM1, except that heterogeneous residual variances replaced the homogenous residual variance. Similarly to a previous report [40], the residual variance in CG $i$ was exponentially regressed on the estimated CG effect of $\hat{\theta }_{i}$ as: $\sigma _{{e_{i} }}^{2} = {\text{exp}}\left( {d_{0} + d_{1} \hat{\theta }_{i} } \right)$, where $d_{0}$ and $d_{1}$ are the intercept and regression coefficients for fitting the residual variance [41]. All variance components were estimated using the average-information restricted maximum likelihood (REML) method implemented in the AIREMLF90 software [35, 36]. The optimal RNM for each trait was chosen based on the AIC values.

### Heritabilities and genetic correlations between environments

The heritability for CG $i$ was calculated as follows [38]:

$$h_{i}^{2} = \frac{{\hat{\sigma }_{{u_{i} }}^{2} }}{{\sum \hat{\sigma }_{{n_{i} }}^{2} + \hat{\sigma }_{e}^{2} }},$$

where $\hat{\sigma }_{{u_{i} }}^{2}$ is the estimate of the additive genetic variance, which was computed as $\hat{\sigma }_{{u_{i} }}^{2} = \hat{\sigma }_{{a_{0} }}^{2} + 2\hat{\sigma }_{{a_{0} a_{1} }} \hat{\theta }_{i} + \hat{\sigma }_{{a_{1} }}^{2} \left( {\hat{\theta }_{i} } \right)^{2}$, and the denominator is the estimate of the phenotypic variance, with $\sum \hat{\sigma }_{{n_{i} }}^{2} = \sum \hat{\sigma }_{{n_{0} }}^{2} + 2\hat{\sigma }_{{n_{0} n_{1} }} \hat{\theta }_{i} + \hat{\sigma }_{{n_{1} }}^{2} \left( {\hat{\theta }_{i} } \right)^{2}$, where $n$ refers to the random effects fitted for each trait (see Table 1). For RNM2, the component $\hat{\sigma }_{e}^{2}$ was calculated as $\hat{\sigma }_{{e_{i} }}^{2} = {\text{exp}}\left( {d_{0} + d_{1} \hat{\theta }_{i} } \right)$.

The genetic correlation for a trait between CG $i$ and $i^{\prime}$ ($r_{{ii^{\prime}}}$) was calculated as follows:

$$r_{{ii^{\prime}}} = \frac{{\hat{\sigma }_{{u_{{ii^{\prime}}} }} }}{{\sqrt {\hat{\sigma }_{{u_{i} }}^{2} \hat{\sigma }_{{u_{{i^{\prime}}} }}^{2} } }},$$

where $\hat{\sigma }_{{u_{{ii^{\prime}}} }}$ is the estimate of the covariance of additive genetic effects between CG $i$ and $i^{\prime}$, which was computed as $\hat{\sigma }_{{u_{{ii^{\prime}}} }} = \hat{\sigma }_{{a_{0} }}^{2} + \hat{\sigma }_{{a_{0} a_{1} }} \hat{\theta }_{i} + \hat{\sigma }_{{a_{0} a_{1} }} \hat{\theta }_{{i^{\prime}}} + \hat{\sigma }_{{a_{1} }}^{2} \hat{\theta }_{i} \hat{\theta }_{{i^{\prime}}}$. Genetic correlation estimates were calculated only for the optimal RNM used for each trait (chosen based on the AIC values).

### Accuracy and correlations of estimated breeding values between traits

The accuracy of genomic estimated breeding values (GEBV) of animal $j$ for a trait was calculated following [42] as:

$$Acc_{j} = \sqrt {1 - \frac{{\widehat{{SE}}_{j}^{2} }}{{\left( {1 + F_{j} } \right)\hat{\sigma }_{{a_{j} }}^{2} }}} ,$$

where $\widehat{{SE_{j} }}$ is the standard error (SE) of the coefficient for the RNM intercept or slope for animal $j$ (square root of the diagonal elements of the inverse of the left-hand side), $F_{j}$ is the inbreeding coefficient of animal $j$, and $\hat{\sigma }_{{a_{j} }}^{2}$ is the variance of the coefficient for the RNM intercept or slope for animal $j$. An estimate of the correlation of GEBV between traits $x$ and $y$ ($r_{{g\left( {xy} \right)}}$) was obtained using the weighted Pearson correlation coefficient of GEBV, as described in [43]:

$$\hat{r}_{{g\left( {xy} \right)}} = \frac{{\sum w_{j} \left( {x_{j} - \bar{x}} \right)\left( {y_{j} - \bar{y}} \right)/\sum w_{j} }}{{\sqrt {\frac{{\sum w_{j} \left( {x_{j} - \bar{x}} \right)^{2} }}{{\sum w_{j} }} \times \frac{{\sum w_{j} \left( {y_{j} - \bar{y}} \right)^{2} }}{{\sum w_{j} }}} }},$$

with $\bar{x} = \frac{{\sum w_{j} x_{j} }}{{\sum w_{j} }}$ and $\bar{y} = \frac{{\sum w_{j} y_{j} }}{{\sum w_{j} }}$, where $x_{j}$ and $y_{j}$ are the GEBV of traits $x$ and $y$, respectively, and $w_{j}$ is the accuracy-based weighting of animal $j$, calculated as $\left( {Acc_{{xj}}^{2} \times Acc_{{yj}}^{2} } \right)/\sqrt {Acc_{{xj}}^{2} \times Acc_{{yj}}^{2} }$. The SE of $\hat{r}_{{g\left( {xy} \right)}}$ was derived as $\sqrt {\left( {1 - \hat{r}_{{g\left( {xy} \right)}}^{2} } \right)/\left( {n - 2} \right)} ,$ where $n$ is the number of selected animals that have GEBV accuracies higher than an empirical value of 0.35 [44, 45].

### Genome-wide association studies and functional analyses

For each trait, the optimal RNM was re-run using the estimated variance components and estimates of the SNP effects for the RNM intercept (${\hat{\mathbf{u}}}_{{\mathbf{0}}}$) and slope (${\hat{\mathbf{u}}}_{{\mathbf{1}}}$) were back-solved following [26] as ${\hat{\mathbf{u}}}_{0} = {\mathbf{IZ^{\prime}}}\left( {{\mathbf{ZIZ^{\prime}}}} \right)^{{ - 1}} {\hat{\mathbf{a}}}_{0}$ and ${\hat{\mathbf{u}}}_{1} = {\mathbf{IZ}}^{1} \left( {{\mathbf{ZIZ}}^{1} } \right)^{{ - 1}} {\hat{\mathbf{a}}}_{1}$, respectively, as implemented in the postGSf90 software [26, 46]. Here, ${\mathbf{Z}}$ is the matrix with the genotypes for each SNP, and ${\hat{\mathbf{a}}}_{0}$ and ${\hat{\mathbf{a}}}_{1}$ are the vectors of GEBV for the RNM intercept and slope, respectively. The proportions of the additive genetic variance that were explained by sliding windows of five adjacent SNPs (sliding genomic windows) computed using the postGSf90 software were reported, and genomic windows were considered to be relevant if they explained 0.5% or more of the total additive genetic variance for a trait for either the intercept or the slope of the RNM [20, 47]. Overlapping relevant windows were concatenated into candidate genomic regions.

We searched all candidate genomic regions in the Pig QTL Database (PigQTLdb, Release 42) [48] in order to query whether they contained previously reported quantitative trait loci (QTL). All known genes within the candidate genomic regions, including the protein-encoding and long non-coding RNAs (lncRNAs), were extracted from the reference pig genome (SusScrofa 11.1; https://uswest.ensembl.org/Sus_scrofa/Info/Index) using the biomaRt R package [49]. Functional enrichment analyses of the candidate genes identified in the previous step were conducted using the g:GOSt function from the g:Profiler web server [50], including the target datasets of the Gene Ontology (GO) biological process [51], Kyoto Encyclopedia of Genes and Genomes (KEGG) pathway [52], and the Human Phenotype Ontology (HPO) term [53]. The default parameters and method of multiple testing correction were used for computing *P* values and a threshold of 0.05 was set. We further illustrated the related biological processes of the candidate genes identified for each trait using the ClueGO software [54].

## Results

### Descriptive statistics of phenotypes and environmental descriptors

Descriptive statistics for the reproduction, growth, and body composition traits after quality control are in Table 1. In total, 186,189 records for TNB, 185,824 records for NBA, and 8164 records for NW were available. For the four growth and body composition traits, the number of phenotypic records ranged from 20,149 (MD) to 101,541 (OW). Up to 474 CG were defined for TNB and NBA, while the number of CG for the other traits ranged from 75 (WW) to 256 (OW). The statistical models used are in Table 1, based on selection of the recorded fixed and prospective random effects [see Additional file 1: Table S2]. The quadratic effects of farrowing age (for NW) and weaning age (for WW and BF) were non-significant and thus, were not included in the final models for these specific traits. All three random effects (i.e., animal, permanent environment, and common litter) were included for TNB, NBA, and NW, but only animal and common litter effects were included for WW, OW, MD, and BF. The density distributions of the estimates of CG effects are in Additional file 2: Fig. S1.

### Reaction norm models and G × E interaction

Estimates of variance components (Table 2) and their corresponding SE [see Additional file 1: Table S3] were obtained for all seven traits with both RNM1 and RNM2. Based on the AIC values, the best RNM differed by trait (Table 2): for TNB, NBA, WW, and OW, the model with a heterogeneous residual variance (RNM2) provided the best fit, while the model with a homogenous residual variance (RNM1) was best for NW, MD, and BF. However, differences in AIC between RNM1 and RNM2 were small for NW, WW, and BF. Differences in estimates of additive genetic variance components for either the RNM intercept or slope between the homogenous and heterogeneous models were small for all traits, except for OW, for which the heteroscedastic model resulted in smaller variance component estimates.

**Table 2:** Estimates of variance components for the intercept and slope for all the models with homogenous (RNM1) and heterogeneous (RNM2) residual variances

Traits	RNM1						RNM2						AIC^a^
	*a*		*pe*		*ce*		*a*		*pe*		*ce*		
TNB^b^	1.0325	0.3011	0.9173	0.3490	0.1146	0.0327	1.0024	0.2533	0.7903	0.1294	0.1132	0.0199	950,276.38
	0.7150	0.1718	0.9999	0.1328	0.9999	0.0932	0.6020	0.1766	0.9989	0.0212	0.9976	0.0035	948,339.79
NBA	0.8056	0.1612	0.7395	0.2975	0.0913	0.0203	0.8014	0.1620	0.6462	0.1199	0.0882	0.0185	936,031.17
	0.4290	0.1754	0.7592	0.2076	0.3627	0.0344	0.4247	0.1816	0.3273	0.2078	0.3178	0.0382	935,390.49
NW	0.2806	− 0.0360	0.1117	− 0.0289	0.1428	− 0.0902	0.2804	− 0.0353	0.1005	0.0164	0.1447	− 0.0892	38,228.143
	− 0.9935	0.0047	− 0.3316	0.0679	− 0.9999	0.0570	− 0.9866	0.0046	0.1839	0.0788	− 0.9999	0.0550	8228.26
WW	0.1988	0.0415	−	−	0.9854	− 0.0040	0.1348	− 0.0184	−	−	0.9831	− 0.0014	100,784.49
	0.5099	0.0334	−	−	− 0.5599	0.0001	− 0.3686	0.0185	−	−	− 0.2317	0.0000	100,780.17
OW	102.570	31.4820	−		32.7150	0.7261	56.9330	8.9481	−	−	33.0810	0.3088	790,691.03
	0.9776	10.110	−	−	0.9915	0.0164	0.9423	1.5838	−	−	0.9985	0.0029	790,366.96
ND	10.5220	0.4543	−	−	3.4842	1.0041	10.3670	0.8726	−	−	3.4046	1.0458	127,475.18
	0.9409	0.0222	−	−	9989	0.2900	0.9855	0.0756	−	−	0.9977	0.3227	127,497.83
BF	7.3740	0.5134	−	−	1.2345	− 0.2079	7.3650	0.5051	−	−	1.2332	− 0.2083	109,070.95
	0.5726	0.1090	−	−	− 0.9994	0.0351	0.5668	0.1078	−	−	− 0.9937	0.0356	109,072.95

TNB, total number of piglets born; NBA, number of piglets born alive; NW, number of piglets weaned; WW, weaning weight (kg); OW, off-test weight (kg); MD, ultrasound muscle depth (mm); BF, ultrasound backfat thickness (mm)

*a*, animal (additive genetic) effect; *pe*, animal permanent environmental effect across parities; *ce,* litter effect

^a^The two Akaike Information Criterion (AIC) values are referred to RNM1 and RNM2, respectively

^b^In the 2 × 2 block between every trait and random effect, the diagonals, upper triangular and lower triangular represent additive genetic variance, covariance and genetic correlation for the intercept and slope coefficients, respectively. The residual variances for both models (RNM1 and RNM2) are in Additional file 1: Table S3

Significant additive genetic variances for RNM slope were observed for TNB, NBA, and OW (*P* < 0.05 based on one-tailed *t*-test). Based on estimates of the variance components of the additive genetic effects, the estimate of the genetic correlation between the RNM intercept and slope were moderate for TNB (0.602 ± 0.024), NBA (0.425 ± 0.028), WW (− 0.368 ± 0.021), and BF (0.573 ± 0.196), but large for NW, OW, and MD (> 0.94).

**Fig. 1:**
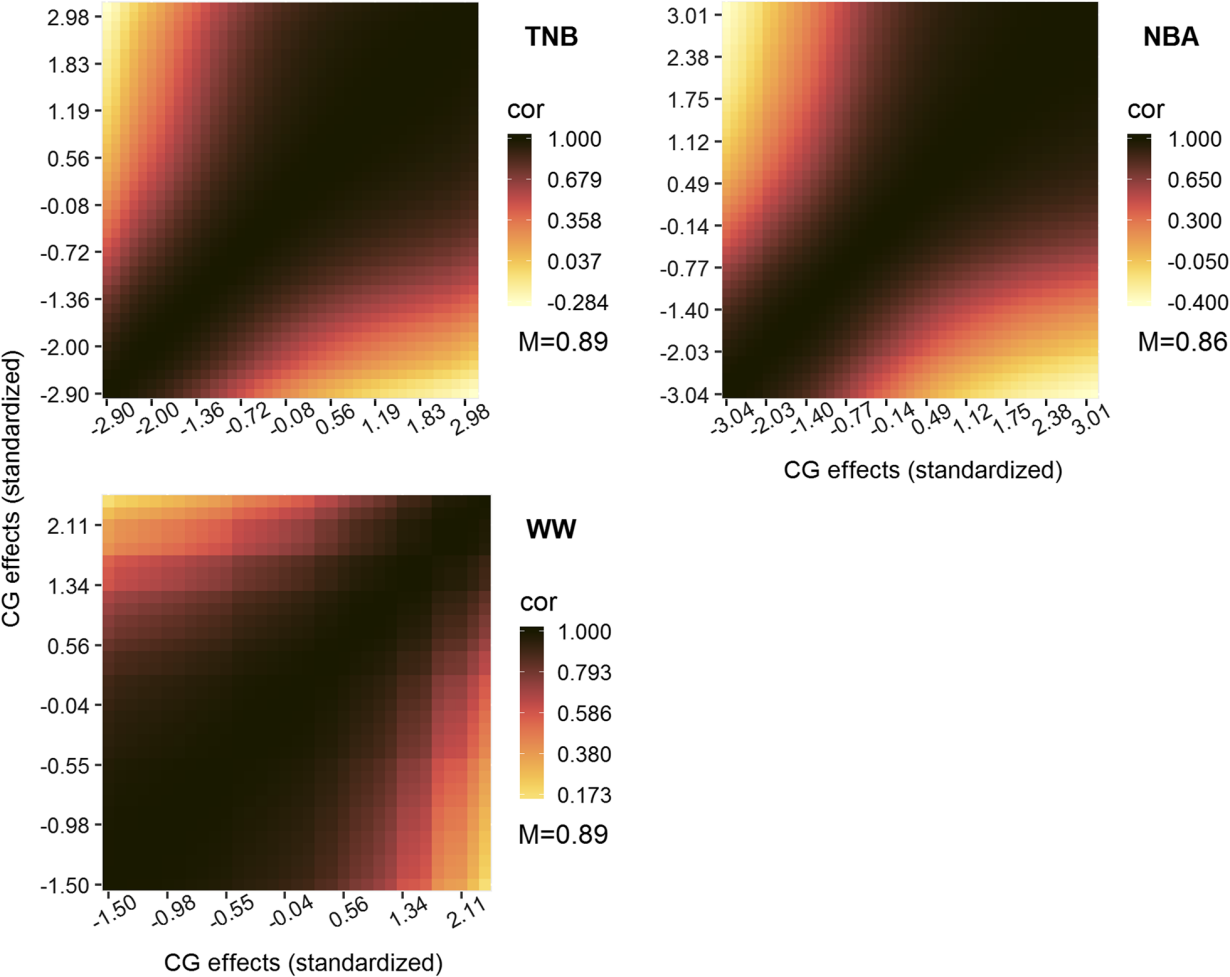
Estimates of genetic correlations across environmental gradients. Pearson correlation coefficients (cor) are represented by colors with the mean values (M) shown below; please note that different scales of color were used according to trait; TNB: total number of piglets born; NBA: number of piglets born alive; WW: weaning weight (kg)

Estimates of genetic correlations for each trait between levels of environmental gradients are shown in Fig. 1. Estimates of the genetic correlation for TNB (average = 0.89) and NBA (average = 0.86) decreased gradually as the difference between environmental gradients increased, as expected. In fact, the correlation estimates were even negative (− 0.28 and − 0.40 for TNB and NBA, respectively) between the first and second-plus-third tertile of the environmental gradients. For WW, lower genetic correlation estimates (average of 0.89 and minimum of 0.16) were also observed between the first-plus-second and third tertile of environmental gradients. However, strong genetic correlation estimates (close to 1.0) across environmental gradients were observed for the other traits (NW, OW, MD, and BF; [see Additional file 2: Fig. S2]). Therefore, we concluded that TNB and NBA are substantially and WW moderately affected by G × E interactions.

### Estimates of heritabilities and breeding values across environments

**Fig. 2:**
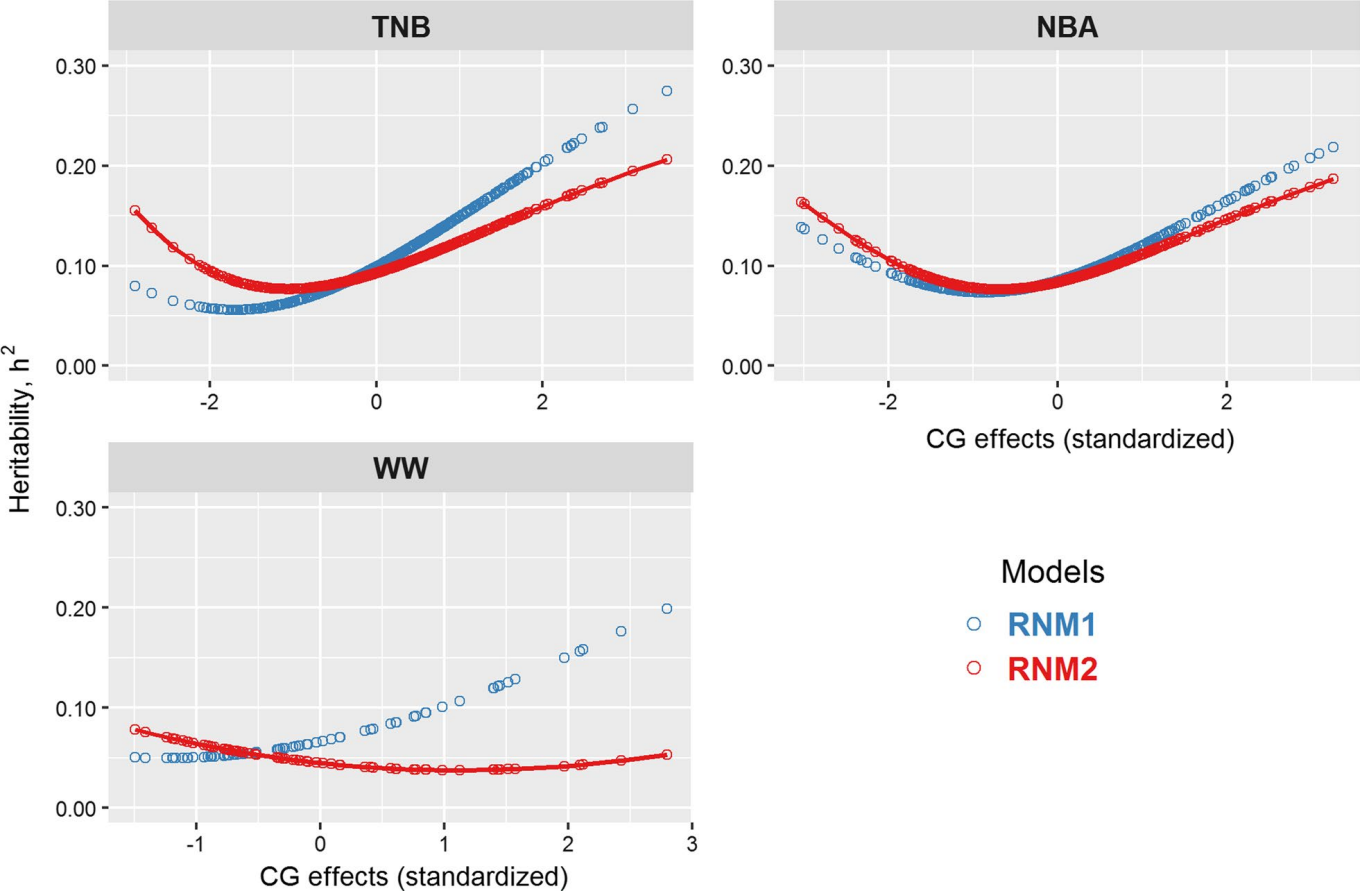
Heritability estimates using reaction norm models with homogenous (RNM1) and heterogeneous (RNM2) residual variances. Respective optimal RNM for each trait are marked by the solid lines; TNB: total number of piglets born; NBA: number of piglets born alive; WW: weaning weight (kg)

For the three traits with significant G × E interactions (TNB, NBA, and WW), estimates of heritability across environments are shown in Fig. 2. The SE of the variance component estimates are in Additional file 1: Table S3. Both TNB and NBA showed similar patterns of heritability estimates along the environmental gradients, i.e., first decreasing, under the worst environmental conditions, and then increasing under better conditions. In addition, clear differences in the magnitude of the heritability estimates between RNM1 and RNM2 were only observed for the extreme environmental conditions. For WW, using RNM1 or RNM2 generated an opposite trend in the heritability estimates when environmental conditions increased (i.e., heritability estimates increased from 0.05 to 0.20 across environmental conditions for RNM1 but slightly decreased from 0.08 to 0.05 for RNM2.

**Fig. 3:**
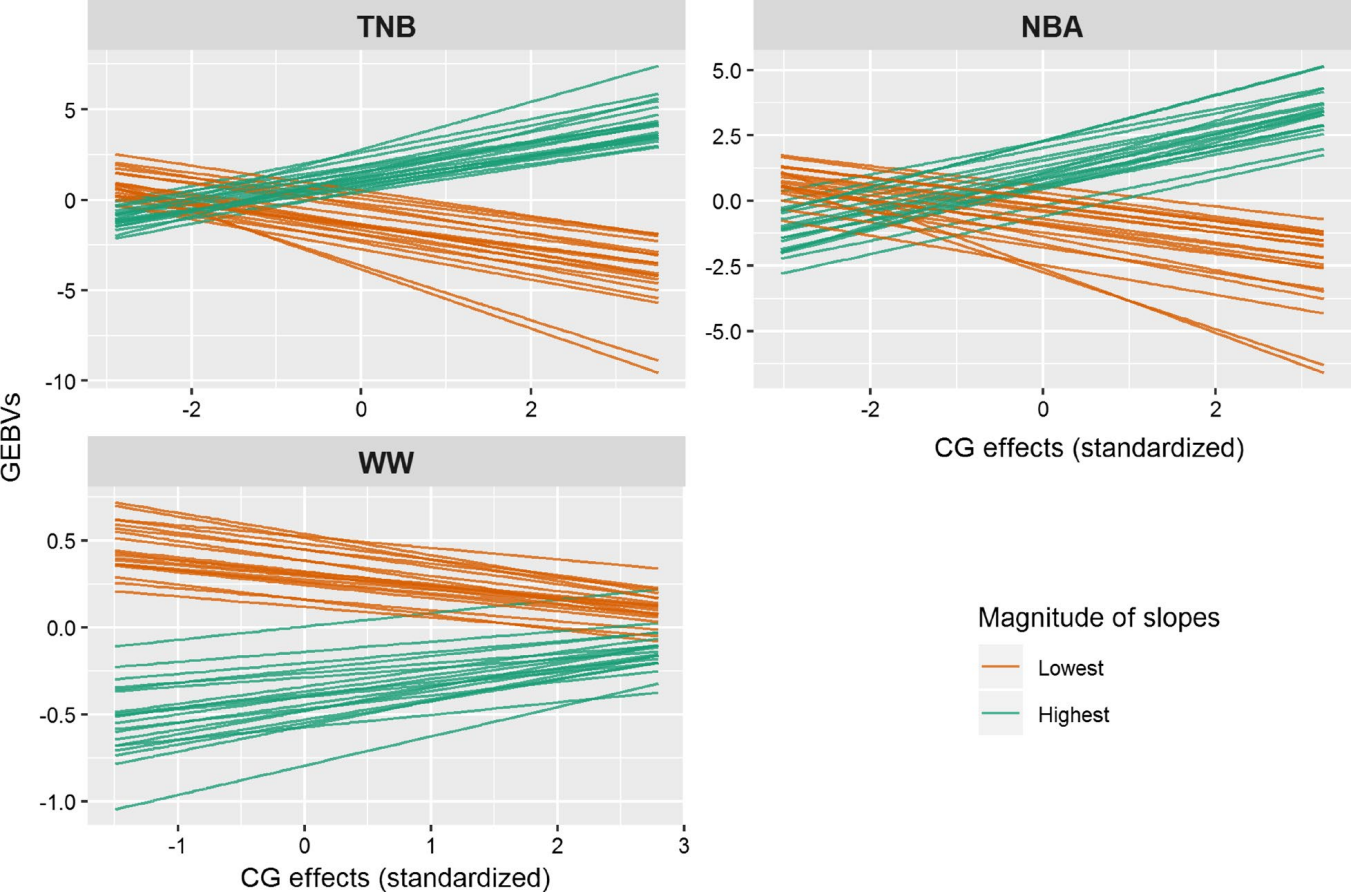
Genomic estimated breeding values (GEBV) of the three traits with clear G×E interactions for 20 sires with the highest and lowest GEBV for reaction norm slopes. TNB: total number of piglets born; NBA: number of piglets born alive; WW: weaning weight (kg).

Among the animals that had more than 30 offspring (i.e., more accurate GEBV with progeny distributed across multiple environmental gradients), Fig. 3 shows the GEBV for the 20 sires with the highest or lowest GEBV for the RNM slope across environments. These sires had the lowest accuracies for WW, with a mean (± SD) of 0.64 ± 0.07 for the GEBV of the RNM intercept and of 0.38 ± 0.06 for the GEBV of the slope. Re-rankings of animals were clearly observed for both TNB and NBA when changing from the worst to the best environmental conditions. A trend towards re-ranking was also observed for WW under the best environmental conditions. Furthermore, we selected all the animals that had more than 10 offspring and a GEBV accuracy for each trait higher than 0.30, and calculated the Spearman’s rank correlation of GEBV between three representative environmental conditions (i.e., ~ 15, 50, and 85% quantiles of environmental gradient, respectively) [see Additional file 1: Table S4]. For TNB and NBA, we observed a moderate and low Spearman’s rank correlation of GEBV between the worst and medium environments (0.614 and 0.669), and between the worst and best environments (0.298 and 0.319), respectively. For WW, the lowest Spearman’s rank correlation of GEBV, i.e. 0.93, was between the worst and best environmental conditions.

For completeness, we also calculated the estimates of heritabilities and the GEBV across environments and performed GWAS for the four traits that did not show significant G × E interactions (NW, OW, MD, and BF). These results are described in Additional file 3 and presented in Additional file 1: Tables S5 and S6 and in Additional file 2: Fig.s S3 to S6.

### Correlations of GEBV between traits

**Fig. 4:**
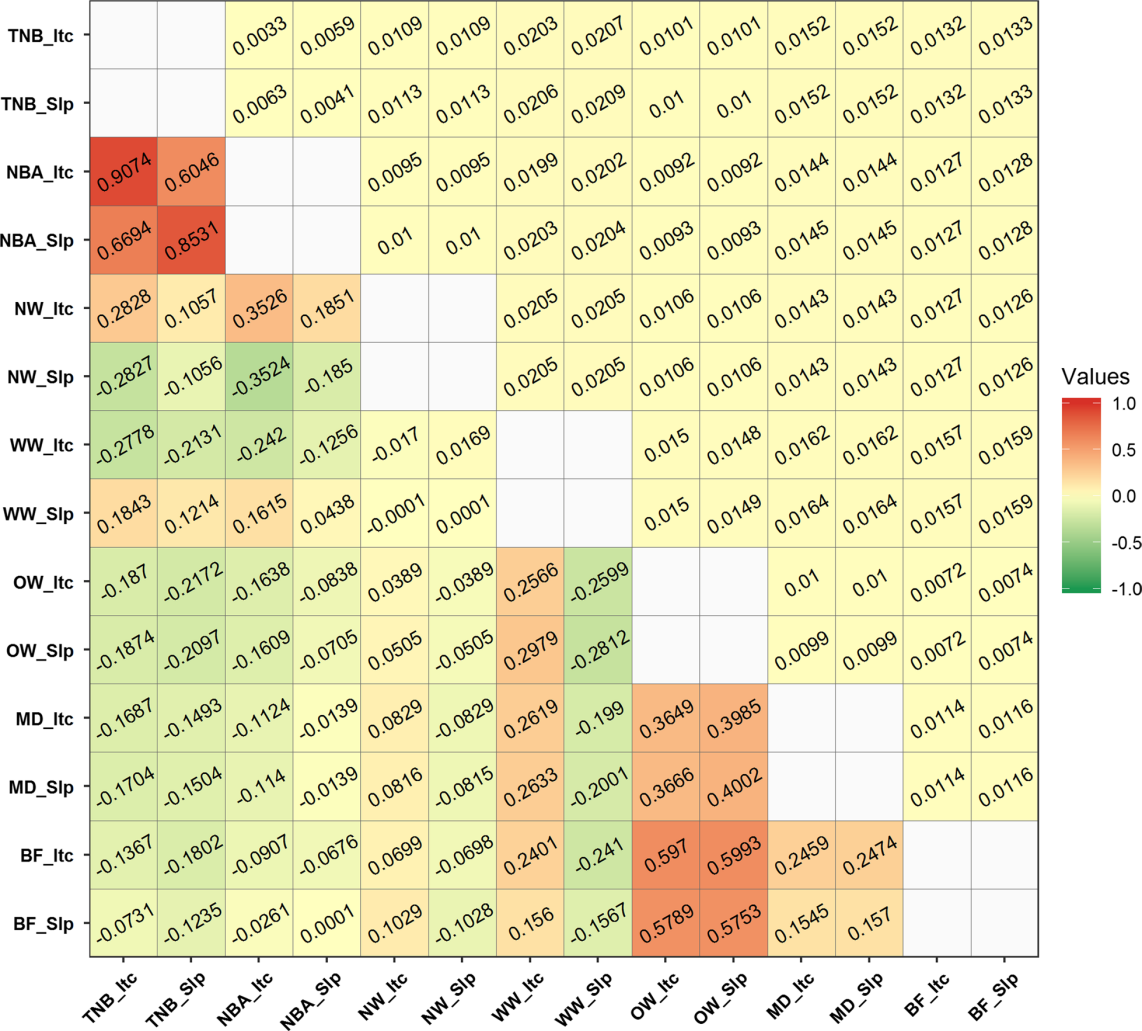
Correlations of genomic estimated breeding values (lower triangle with standard errors in the upper triangle) between traits for reaction norm intercepts and slopes. The x-axis and y-axis are the concatenation of traits and RNM items, for instance, “TNB_Itc” and “TNB_Slp” represent the RNM intercept (Itc) and slope (Slp) of the “TNB” trait, respectively. Within each trait, the real value of the genetic correlation between RNM intercept and slope is not shown here; TNB: total number of piglets born; NBA: number of piglets born alive; NW: number of piglets weaned; WW: weaning weight (kg); OW: off-test weight (kg); MD: ultrasound muscle depth (mm); BF: ultrasound backfat thickness (mm)

Before estimating the correlations of GEBV between traits, we investigated the accuracies of GEBV for the RNM intercept and slope for each trait [see Additional file 1: Table S7]. For the RNM intercept, the highest and lowest average accuracies of GEBV for all animals were observed for OW [0.649, 95% confidence interval (CI) of 0.648–0.649] and NW (0.393, 95% CI of 0.391–0.395), respectively. The RNM slopes had relatively lower accuracies of GEBV that ranged from 0.292 (95% CI of 0.291–0.293) for WW to 0.606 (95% CI of 0.605–0.606) for OW. For the estimation of the correlation between TNB and WW, the number of selected animals (N = 2252) was the smallest [see Additional file 1: Table S8]. The estimated weighted Pearson correlation between RNM intercepts and slopes of traits are shown in Fig. 4.

Among the seven traits analyzed, the highest positive correlations of GEBV were between TNB and NBA, with values of 0.91 for the RNM intercept and 0.85 for the RNM slope. Both TNB and NBA had negative correlations with NW for the GEBV of the RNM slope (0.11–0.35) and with WW for the GEBV of the RNM intercept (0.13–0.28). Also, TNB and NBA had negative correlations with growth and body composition traits for both the RNM intercept and slope (OW, MD, and BF). The RNM intercept and slope of WW had positive (~ 0.28) and negative (~ 0.27) correlations with OW, respectively. The GEBV correlations ranged from 0.15 to 0.25 between MD and BF for the RNM intercept and slope. However, we observed moderate and positive GEBV correlations of OW with MD (0.36–0.40) and BF (0.57–0.60). Between the intercept-by-intercept and slope-by-slope comparisons, the GEBV correlations were in opposite directions for 12 of the comparisons, such as for TNB with NW, WW with OW, and WW with MD (Fig. 4).

### Genome-wide association studies

**Fig. 5:**
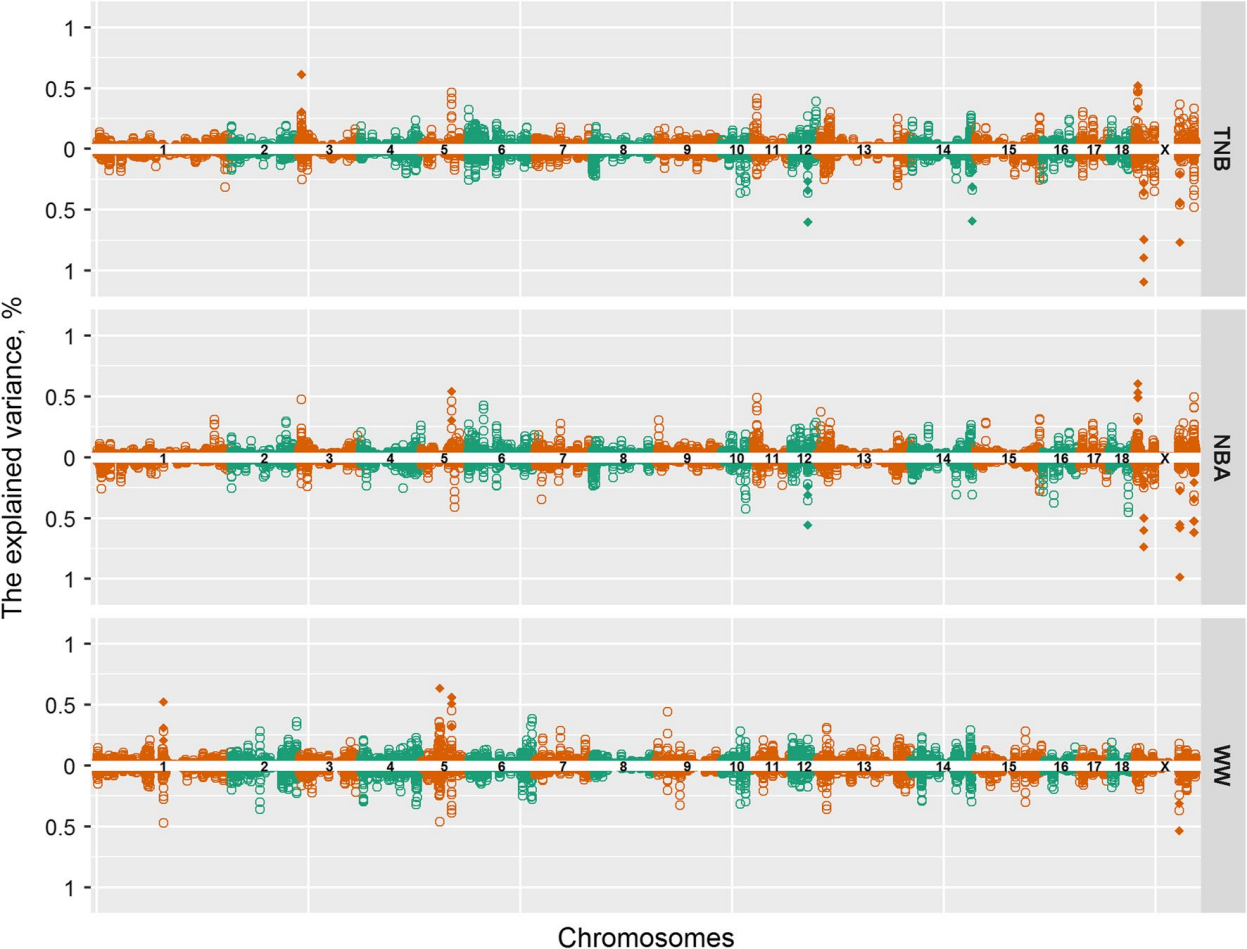
Miami plots for the proportion of the total additive genetic variance explained by 5-SNP sliding genomic windows. The intercept and slope terms of the reaction norm model are placed on the upper and lower arms of the y-axis, respectively; each open dot represents a SNP, while all SNPs within the relevant genomic windows are denoted as solid diamonds; TNB: total number of piglets born; NBA: number of piglets born alive; WW: weaning weight (kg)

**Fig. 6:**
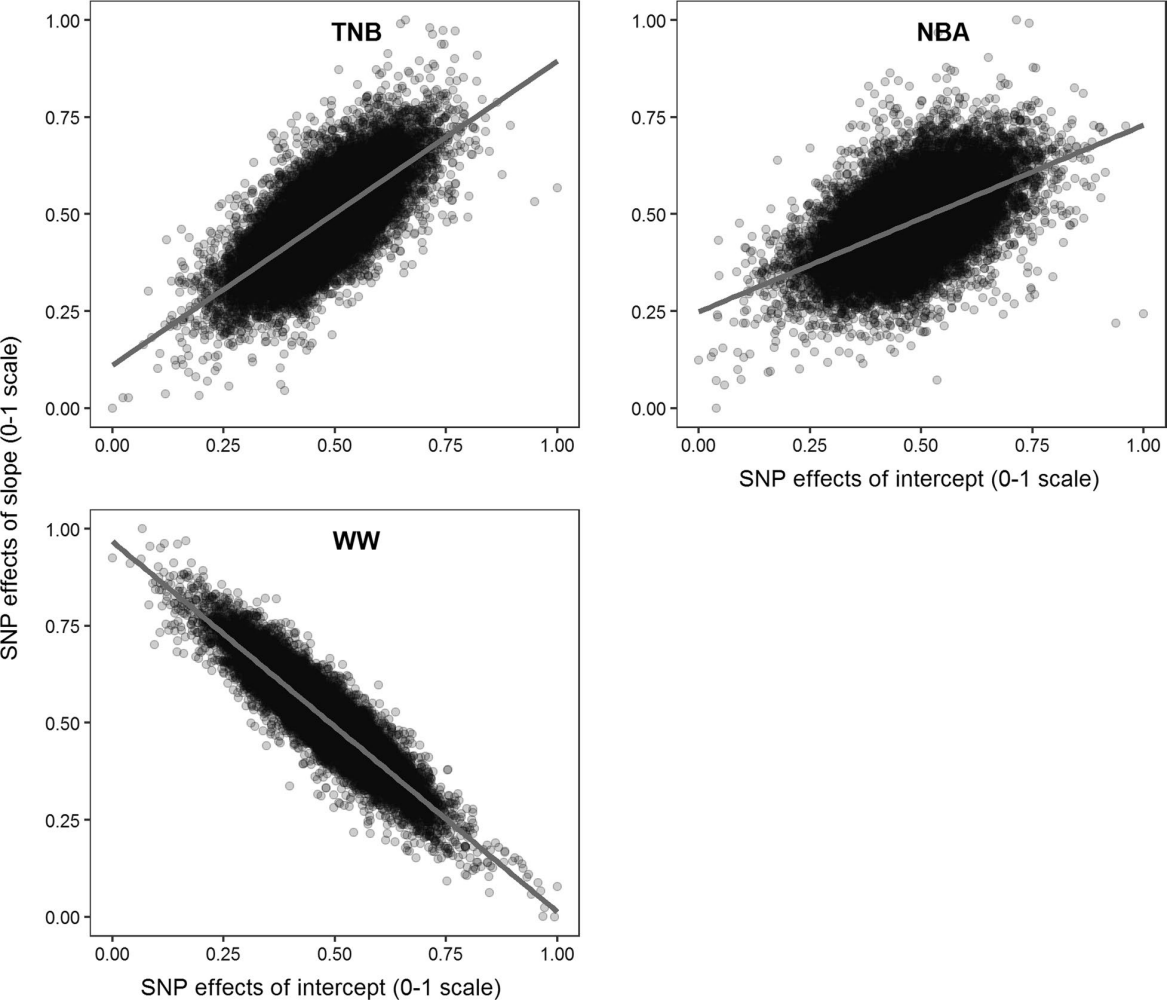
Estimates of correlation between SNP effect estimates for the reaction norm intercept and slope. TNB: total number of piglets born; NBA: number of piglets born alive; WW: weaning weight (kg)

For TNB, NBA, and WW, 27 relevant 5-SNP windows were identified, which were concatenated into 16 genomic regions with 65 SNPs, distributed on five autosomes and the X chromosome, each explaining 0.5% or more of the additive genetic variance (Table 3 and Fig. 5). Among these, four genomic regions on the *Sus scrofa* (SSC) chromosome X (SSCX) and one window on SSC12 overlapped between TNB and NBA. For TNB and NBA, no genomic windows were significant for both the RNM intercept and slope, and the numbers of relevant genomic windows were larger for the slope than for the intercept (i.e., 6 versus 2 for TNB, and 10 versus 4 for NBA). In contrast, for WW more relevant genomic windows were found for the RNM intercept (4/5) than for the RNM slope (1/5). More than half of the relevant genomic windows were located on SSCX for TNB (5/8) and NBA (12/14), while only one genomic window (1/5) was located on SSCX for WW. Based on the estimated SNP effects (Fig. 6), the correlation between the RNM intercept and slope was moderately positive for TNB and NBA (0.72 and 0.54, respectively) but negative for WW (− 0.92).

**Table 3:** Relevant genomic windows with the explained genetic variances and associated candidate genes

Trait	Genomic window^a^		Variance (%)^b^		Candidate genes
	Chr	Positions (bp)	Int	Slo	
TNB	SSC3	5,934,183–5,978,000	*0.61*	0.00	*TRRAP*
	SSC12	37,811,479–37,872,427	0.27	*0.60*	ENSSSCG00000039473
	SSC14	134,570,084–134,625,180	0.06	*0.59*	None
	SSCX	7,243,129–7,307,313	*0.52*	0.16	*MID1*
		19,844,004–19,985,584	0.11	*0.89*	*ACOT9*, *SAT1*, *APOO*, *CXorf58*
		19,885,476–20,012,307	0.15	*1.09*	
		19,911,826–20,026,618	0.10	*0.74*	
		91,905,396–92,010,688	0.37	*0.77*	*TRPC5*
NBA	SSC5	69,872,903–70,055,268	*0.54*	0.00	ENSSSCG00000000769, *MICAL3*
	SSC12	37,811,479–37,872,427	0.20	*0.56*	ENSSSCG00000039473
	SSCX	7,198,352–7,278,758	*0.53*	0.20	*MID1*
		7,213,512–7,293,780	*0.60*	0.15	
		7,243,129–7,307,313	*0.61*	0.07	
		19,844,004–19,985,584	0.01	*0.60*	*ACOT9*, *SAT1*, *APOO*
		19,885,476–20,012,307	0.02	*0.74*	
		91,889,310–91,994,567	0.14	*0.57*	*TRPC5*
		91,905,396–92,010,688	0.23	*0.98*	
		91,966,658–92,025,442	0.13	*0.55*	
		91,979,985–92,034,525	0.10	*0.58*	
		122,118,491–122,263,709	0.40	*0.52*	*MAMLD1*, *MTM1*
		122,149,267–122,298,170	0.41	*0.52*	
		122,194,006–122,365,194	0.49	*0.62*	
WW	SSC1	141,889,209–142,070,404	*0.52*	0.47	*UBE3A*, ENSSSCG00000050514*
	SSC5	41,461,124–41,616,882	*0.64*	0.46	*PKP2*, *YARS2*, ENSSSCG00000000530
		69,770,951–70,055,268	*0.51*	0.37	*ATP6V1E1*, *BCL2L13*, ENSSSCG00000000769, *MICAL3*
		69,872,903–70,106,805	*0.56*	0.39	
	SSCX	91,438,755–91,518,334	0.18	*0.54*	*DCX*

TNB, total number of piglets born; NBA, number of piglets born alive; WW, weaning weight (kg)

^a^Genomic windows are defined by the five adjacent SNPs that explained 0.5% or more of the total additive genetic variance; Chr, chromosome; positions refer to Sscrofa11.1

^b^Explained genetic variances in percent for intercept (Int) and slope (Slo) by five adjacent SNPs; explained variances with ≥ 0.5% are denoted in italics.

Candidate genes are represented by the gene symbol when available, otherwise by the Ensembl gene ID. The long noncoding RNA genes are marked by marked by an asterisk (^*^)

### Functional investigation of the identified relevant genomic regions

We searched all the relevant genomic regions using the PigQTLdb and found that most of them (13/16) were located within previously reported QTL for the same (or a biologically associated) trait (Table 4). Among these, previously reported QTL for litter size, number of mummified pigs, plasma FSH (follicle-stimulating hormone) concentration, and health-associated QTL (such as pathogen susceptibility and immunity response) were associated with the relevant genomic regions for TNB and NBA identified in this study. The four relevant genomic regions for WW were located within previously reported QTL for traits such as WW, birth weight, and immunity response. The relevant genomic regions included 19 protein-encoding and one lncRNA genes (Table 3 and Additional file 1: Table S9). In addition to the five candidate genes on SSCX that were shared between TNB and NBA, two additional candidate genes were found on SSCX for NBA. Four relevant genomic regions on SSCX were jointly supported by three or more overlapping sliding windows, including the regions for TNB from 19,844,004 to 20,026,618 bp, and for NBA from 7198,352 to 7,307,313 bp, from 91,889,310 to 92,034,525 bp, and from 122,118,491 to 122,365,194 bp.

**Table 4:** Previously reported quantitative trait loci (QTL) for related traits within candidate genomic regions

Trait	Genomic region (bp)^a^	Related QTL (number of reports)
TNB	SSC3: 5,934,183–5,978,000	Litter size (1), teat number (1), corpus luteum number (1), body weight at birth (1)
	SSC12: 37,811,479–37,872,427	Teat number (7), body weight at birth (1), pathogen susceptibility (2), immunity response (3)
	SSC14: 134,570,084–134,625,180	Teat number (3), number of mummified pigs (3), body weight at birth (1), maternal infanticide (1), immunity response (1)
	SSCX: 7,243,129–7,307,313	Teat number (1)
	SSCX: 19,844,004–20,026,618	None
	SSCX: 91,905,396–92,010,688	Plasma FSH concentration (1), testicular parenchyma color (1), immunity response (1)
NBA	SSC5: 69,872,903–70,055,268	Number of stillborn (1), teat number (1), immunity response (1)
	SSC12: 37,811,479–37,872,427	Teat number (7), body weight at birth (1), pathogen susceptibility (2), immunity response (3)
	SSCX: 7,198,352–7,307,313	Teat number (1)
	SSCX: 19,844,004–20,012,307	None
	SSCX: 91,889,310–92,034,525	Plasma FSH concentration (1), testicular parenchyma color (1), immunity response (1)
	SSCX: 122,118,491–122,365,194	None
WW	SSC1: 141,889,209–142,070,404	Teat number (3), body weight at weaning (3), body weight at birth (1), immunity response (5), melanoma susceptibility (2)
	SSC5: 41,461,124–41,616,882	Number of stillborn (1), teat number (1), immunity response (1)
	SSC5: 69,770,951–70,106,805	Number of stillborn (1), teat number (1), immunity response (1)
	SSCX: 91,438,755–91,518,334	Plasma FSH concentration (1), testicular parenchyma color (1), immunity response (1)

TNB, total number of piglets born; NBA, number of piglets born alive; WW, weaning weight (kg)

^a^Genomic regions are concatenated by the overlapped genomic windows shown in Table 3, and the positions are referred to Sscrofa11.1

No significantly enriched GO biological process or KEGG pathway was revealed for any of the traits in the functional enrichment analyses. Thus, we investigated the involved biological processes for each candidate gene [see Additional file 2: Fig. S6]. Thirteen of the 20 candidate genes were involved in one or more biological processes and some of these genes have positive biological implications, such as the GO terms of “Negative regulation of microtubule depolymerization” and “Manganese ion transport” for TNB and NBA. Furthermore, five HPO terms of the penile hypospadias (HP:0003244), blind vagina (HP:0040314), glandular hypospadias (HP:0000807), penoscrotal hypospadias (HP:0000808), and X-linked recessive inheritance (HP:0001419) were suggested for the three *MAMLD1* (*mastermind like domain containing 1*), *MTM1* (*myotubularin 1*), and *MID1* (*midline 1*) genes that were associated with NBA [see Additional file 2: Fig. S7].

## Discussion

Although the existence of G × E interactions for quantitative traits in livestock has been widely recognized for many decades [55], significant progress in genetic and genomic evaluations of GxE interactions has emerged only during recent years, mainly due to advances in genomic technologies and analytic methods [9, 10]. In an early study, Schinckel et al*.* [56] reared multiple genetic pig populations under different environments and provided conclusive evidence that considering G × E interactions is an important factor to be considered when genetically evaluating pigs. By comparing the performance of the progeny of boars from three terminal lines, the genotype-by-feeding-level interactions were evaluated for average daily feed intake, growth rate, feed conversion ratio, and backfat [57]. These studies on G × E interactions in pigs, together with those of Knap et al. [58], Wallenbeck et al. [16], Brandt et al. [59], Li et al. [17], Rosé et al. [60], and Godinho et al. [61] were conducted using well-known pig breeds, progeny groups of sires, or purebred-crossbred populations to represent different genomic backgrounds. However, the inter-individual genetic differences within breeds or progeny groups were not appropriately accounted for in these studies.

Using the pedigree-based relationship matrix (${\mathbf{A}}$), Sevillano et al. [62], Godinho et al. [63], and Gourdine et al. [15] evaluated GxE interactions for economically important traits in pigs under various environmental conditions, including photoperiod regimes, ambient temperature, and feed composition. In 2014, the genomic relationship matrix (${\mathbf{G}}$) based on genome-wide SNPs was first used to evaluate G × E interactions for TNB in pigs [38] and the inclusion of genomic information was found to improve selection accuracy across environments. Subsequently, genetic evaluation of G × E interactions using single-step genomic RNM (i.e., using the hybrid ${\mathbf{H}}$ matrix instead of ${\mathbf{G}}$) was proposed for growth traits in response to heat stress in purebred nucleus and commercial crossbred pigs [12]. Recently, G × E interactions have also been studied for two growth [14] and three reproduction [13] traits in pigs using single-step genomic RNM, in which environmental gradients were quantified based on the estimated average performances of CG and covariates derived from weather records, respectively. In our study, we used the average performance of CG as environmental gradients and comprehensively evaluated G × E interactions using the single-step genomic RNM for seven reproduction, growth, and body composition traits in Large White pigs, which represents one of the most commonly raised maternal line breeds. Analysis of multiple economically important traits using the same statistical method enabled a more straightforward comparison of G × E interactions between traits. In addition, we incorporated SNPs that are located on the X chromosome in the genomic analyses, which are frequently ignored in studies of this nature. However, we acknowledge that alternative methods for incorporating SSCX markers in a ssGBLUP setting should be evaluated in future studies.

### Reaction norm models

The RNM is an effective approach for the evaluation of G × E interactions with continuous environmental descriptors, where animals raised in different environments can be connected to each other using pedigree and/or genomic information [2]. Zhang et al. [23] studied G × E interactions for reproduction traits in Holstein cattle and indicated that the use of the ${\mathbf{H}}$ matrix in RNM can improve prediction accuracy, as it is commonly done in other studies in pigs [12, 14, 22]. Thus, in this study, we also used the RNM coupled with the ${\mathbf{H}}$ matrix. Since inheritance patterns differ between the autosomes and the X chromosome, specific approaches (such as different genotyping coding rules) must be used to include SSCX SNPs in the construction of genomic-based relationship matrices [28, 64, 65]. In addition to the dosage compensation effect in females [30], the different number of copies of the X chromosome between males and females complicates the calculation of individual relationships. However, Su et al. [28] found that exclusion of X-chromosome markers had only a small effect on the accuracy of GEBV for 15 traits included in the Nordic Total Merit index of Nordic Holstein bulls. In our study, we evaluated exclusion of either the X-chromosome markers (N = 2344, 4.2%) or the genotyped males (N = 1669) for the two traits with significant G × E interactions (TNB and NBA) and we did not find significant changes in heritability estimates or GEBV accuracies when excluding the X-chromosome markers from the analyses [see Additional file 2: Fig. S8]. However, construction of a separate relationship matrix that is based only on SNPs from the X-chromosome is recommended for future studies. The small number of SNPs on the X-chromosome usually results in singular matrices, which is a challenge for ssGBLUP analyses [61]. Therefore, future studies should investigate modelling of the relationship matrices that include SNPs on the X-chromosome in a ssGBLUP setting, as proposed by Druet and Legarra [61].

Another important issue regarding the evaluation of G × E interactions involves the modelling of homogenous or heterogeneous residual variances between different environmental conditions in RNM. Carvalheiro et al. [20] analyzed G × E interactions for post-weaning weight gain in beef cattle (Nellore, *Bos taurus indicus*) using a comprehensive dataset and suggested that RNM with heterogeneous residual variances (termed heteroscedastic RNM) provided a better fit to the data than a homoscedastic RNM. However, our findings show that, actually, the choice of homogenous or heterogeneous residual variances is trait-dependent because the best fit was obtained with the homoscedastic RNM for three of the seven traits (NW, MD, and UF). The two RNM studies performed on pigs, by Song et al. [14] and Tiezzi et al. [13], only used either homoscedastic or heteroscedastic RNM, respectively, and different models for residual variances were not compared.

### G × E interactions and genetic parameters

Estimates of the variance components for the RNM slope and of the genetic correlations between different environmental conditions can be used to evaluate whether G × E interactions are present or not. In general, estimates of genetic variance of RNM slopes that significantly differed from zero and/or of genetic correlations between different environments that are lower than 0.8 have been proposed as evidence of G × E interactions in livestock [2, 23]. In this context, our study revealed significant G × E interactions for TNB and NBA according to both the estimates of the genetic variance of RNM slopes and of the genetic correlations between environments. However, a possible G × E interaction (or a trend) was also suggested for WW, because low genetic correlation estimates between the first-plus-second and third tertile of environmental gradients were found. G × E interactions were not observed for NW, OW, MD, and BF. With respect to the GEBV, the re-ranking of pigs across environments also support the conclusion of the presence of G × E interactions for TNB, NBA, and WW, but not for the other traits. Overall, our results regarding G × E interactions in pigs are consistent with other reports for TNB and NBA [13, 17]. However, different conclusions were previously reported for BF, including the absence [38] or presence [14, 60, 66] of G × E interactions in pigs. Moreover, Fragomeni et al. [12] observed a significant G × E interaction for body weight at ~ 170 days of age in purebred Duroc but not in crossbred animals. These results suggest that a heterogeneous biological basis underlies the G × E interaction, which may depend on the genetic background of the population studied and/or the type of environment involved.

Heritability estimates vary considerably between environments when taking G × E interactions into consideration. Silva et al. [38] used the average performances of CG as environmental gradients and reported the highest (0.13) and lowest (0.04) heritability estimates for TNB under the best and medium environmental conditions, respectively. Based on the average relative humidity before conception or the average THI index during the pregnancy of sows [13], lower heritability estimates were observed when environmental conditions became more uncomfortable (e.g., too low or too high temperatures), from 0.12 to 0.02 for TNB and from 0.23 to 0.02 for NBA. The patterns of heritability estimates across environments (i.e., curve shapes) observed in our study were similar to those reported in the literature [13, 38]. In addition, our heritability estimate for WW agreed with that reported previously in Large White, Yorkshire, and Landrace pigs (0.01–0.08) [67, 68].

To date, few studies have investigated the genetic relationships between traits when accounting for G × E interactions. According to the GEBV correlations calculated among all the studied traits, we found the highest positive correlations between TNB and NBA for both the RNM intercept and slope, which are similar to the high estimates previously reported [69, 70]. This suggests that the GEBV correlations calculated in this study are reliable. However, only relatively low positive correlations were observed for the RNM intercepts between NW and TNB/NBA in spite of the high estimates (0.76–0.91) previously reported in the Finnish Landrace and Large White populations without considering G × E interaction [69]. In contrast to the moderate negative genetic correlations reported in a previous study [68], OW had relatively low positive and negative correlations with the RNM intercept and slope of WW, respectively. We observed moderate positive correlations of OW, MD, and BF with both the RNM intercept and slope, as reported in previous studies [71]. Furthermore, we observed that the genetic correlations between the RNM intercept and slope were often in opposite directions, which suggests that different relationships exist between average performance and environmental sensitivity of traits.

### Dissection of SNP effects and functional implications

Although several candidate genomic windows were found to be associated with the traits investigated in this study, we believe that this number is probably conservative, because of the stringent setting applied, i.e. the relevant genomic windows were required to explain 0.50% or more of the total additive genetic variance. Alternatively, a less stringent method would be to select the top-N candidate genomic windows according to the magnitude of the explained variance or approximate *P*-value of SNPs, as used in previous studies [13, 14]. However, the candidate genomic regions identified in our study overlapped with previously identified QTL for the same or biologically-related traits. The use of RNM provides an opportunity to distinguish the SNP effects as either environment-robust or environment-sensitive, which is a method that has been commonly used in studies on G × E interactions in livestock (e.g. [13, 14, 20, 23]).

In this study, we observed that most of the candidate genomic windows (> 70%) for TNB and NBA are involved in environmental sensitivity, as they are associated with the RNM slope. In this context, it is important to highlight that these relevant genomic windows were mainly located on SSCX. However, no SNP in the candidate genomic regions for TNB and NBA overlapped with the hundreds of candidate SNPs for the two traits published in a recent similar study in pigs by Tiezzi et al. [13]. A possible reason for this, apart from the small number of candidate SNPs in our study, is that the X chromosome was not included in the study by Tiezzi et al. [13]. Currently, the PigQTLdb includes more than 300 QTL that have been found to be significantly associated with TNB and NBA in pigs [32] but less than 10 of these SNPs are located on SSCX because this chromosome was not commonly included in previous studies. Interestingly, the two candidate genes on SSCX detected in our study, *MID1* and *TRPC5* (*transient receptor potential cation channel subfamily C member 5*), have been reported to be significantly associated with NBA and other reproduction traits in Landrace and Large White pigs [72]. The other two genomic regions located on SSCX could include genes that are functionally involved in litter size traits in pigs, for example, the *MAMLD1* and *MTM1* genes. These genes, together with *MID1*, are known to have a role in reproduction-related phenotypes in humans [73, 74]. Both the observed number of relevant genomic windows and the functional implications of the candidate genes provide further evidence regarding the biological contribution of the X chromosome to TNB and NBA, which also suggests the importance of including the X chromosome in the genetic evaluation of G × E interactions, especially for reproduction traits. As a next step, we will evaluate more sophisticated approaches to include the markers on SSCX in ssGBLUP analyses, compare the use of average performance of contemporary groups and other environmental gradient levels (e.g., temperature, relative humidity), and perform functional genomic analyses to identify the potential causal mutations located in the relevant genomic regions.

Among the five relevant genomic windows found for WW, four and one genomic windows were independently associated with the RNM intercept and slope, respectively. Although little biological evidence is available in the literature on candidate genes for WW, the genomic region on SSCX is closely located to one candidate gene (*TRPC5*) for TNB and NBA. In addition, the relevant genomic regions for WW overlap with multiple previously reported QTL for health-related traits in pigs, including melanoma susceptibility and immune response [75, 76], which represent reasonable links between WW and health-related performance. Furthermore, the genomic region on SSC1 found for WW is supported by a series of previously reported QTL for growth traits in pigs. The candidate gene for WW, *UBE3A* (*ubiquitin protein ligase E3A*), has also been reported to be associated with TNB in pigs [77].

## Conclusions

In this study, we fitted the average performance of contemporary group as the environmental gradient and comprehensively evaluated G × E interactions using the single-step genomic RNM method for reproduction, growth, and body composition traits in Large White pigs. GxE interactions were detected for two reproduction traits (TNB and NBA) and suggested for WW. No G × E interaction was observed for the other four traits (NW, OW, MD, and BF). For the RNM, the choice of homogeneous or heterogeneous residual variances depended on the trait studied. By dissecting the SNPs with different effects across environmental gradient levels, we detected several candidate SNPs, genes, and genomic regions, which contribute to better understand the biological basis of G × E interactions for these traits. Furthermore, our results emphasize the biological contribution of the X chromosome to reproduction traits in pigs, especially regarding their G × E interactions.

## Supplementary Information

**Additional file 1: Table S1.** Summary of the phenotypic records used in this study before data editing and quality control**. Table S2.** Selection of the fixed effects, covariates, and random effects included in the final mixed animal models**. Table S3.** Standard errors of variance components estimates based on the homogenous (RNM1) and heterogeneous (RNM2) residuals**. Table S4.** Spearman’s rank correlations of genomic estimated breeding values across three representative environmental gradients**. Table S5.** Genomic windows with the significantly explained variances and associated candidate genes for the four traits without clear G × E interaction. **Table S6.** Related quantitative trait loci (QTL) within relevant genomic regions for the four traits without clear G × E interaction. **Table S7.** Accuracies and reliabilities of genomic estimated breeding values for all animals. T**able S8.** Sample sizes and accuracies used for calculating the approximate genetic correlations (weighted Pearson coefficients). **Table S9.** Detailed information for the candidate genes found in this study.

**Additional file 2: Fig. S1.** Density distribution of the estimated effects of contemporary groups. **Fig. S2.** Genetic correlations across environmental gradients using the optimal reaction norm models for the four traits without G × E interaction. **Fig. S3.** Heritability estimates using reaction norm models with homogenous (RNM1) and heterogeneous (RNM2) residual variances for the four traits without G × E interaction. **Fig. S4.** Genomic estimated breeding values using the optimal reaction norm models for 20 sires with the highest and lowest reaction norm slopes, respectively (for the four traits without G × E interaction). **Fig. S5.** Miami plots for the proportion of explained variance by 5-SNP sliding genomic windows for the four traits without clear G × E interaction. **Fig. S6.** Biological processes involved for all candidate genes identified in this study. **Fig. S7.** Five enriched terms of Human Phenotype Ontology for the number of piglets born alive (NBA). **Fig. S8.** Heritability estimates for the total number of piglets born (TNB) and number of piglets born alive (NBA) across environmental gradients using three scenarios regarding the X-chromosome markers.

**Additional file 3:** Detailed results for the four traits that did not show significant G × E interactions.

## Data Availability

All the data supporting the results of this study are included in the article and in the Additional files. The raw phenotypic and genotypic data cannot be shared because they are owned by commercial breeding companies and this information is commercially sensitive.
