# Supplementary material for: Genotype-by-environment interactions for reproduction, body composition, and growth traits in maternal-line pigs based on single-step genomic reaction norms
Source: Genet Sel Evol. 2021 Jun 17;53:51. doi: 10.1186/s12711-021-00645-y (PMC8212483; doi:10.1186/s12711-021-00645-y)
Supplement: Supplementary file 1 — Additional file 1: Table S1. Summary of the phenotypic records used in this study before data editing and quality control. Table S2. Selection of the fixed effects, covariates, and random effects included in the final mixed animal models. Table S3. Standard errors of variance components estimates based on the homogenous (RNM1) and heterogeneous (RNM2) residuals. Table S4. Spearman’s rank correlations of genomic estimated breeding values across three representative environmental gradients. Table S5. Genomic windows with the significantly explained variances and associated candidate genes for the four traits without clear G × E interaction. Table S6. Related quantitative trait loci (QTL) within relevant genomic regions for the four traits without clear G × E interaction. Table S7. Accuracies and reliabilities of genomic estimated breeding values for all animals. Table S8. Sample sizes and accuracies used for calculating the approximate genetic correlations (weighted Pearson coefficients). Table S9. Detailed information for the candidate genes found in this study. [file 12711_2021_645_MOESM1_ESM.docx]

**Table S1 Summary of phenotypic records used in this study before the data editing and quality control**

| **Traits**^1^ | **Number of records** | **Number of animals** | **Number of farms distributed** | **Range of years recorded** |
| --- | --- | --- | --- | --- |
| TNB | 186,909 | 71,243 | 30 | 2005 - 2019 |
| NBA | 186,964 | 71,245 | 30 | 2005 - 2019 |
| NW | 8,616 | 3,439 | 3 | 2005 - 2019 |
| WW | 28,261 | 28,261 | 3 | 2010 - 2019 |
| OW | 204,014 | 204,014 | 27 | 2004 - 2019 |
| MD | 21,050 | 21,050 | 4 | 2004 - 2019 |
| BF | 21,051 | 21,051 | 4 | 2004 - 2019 |

^1^ TNB: total number of piglets born; NBA: number of piglets born alive; NW: number of piglets weaned; WW: weaning weight (Kg); OW: off-test weight (Kg); MD: ultrasound muscle depth (mm); BF: ultrasound backfat thickness (mm).

**Table S2 Selection of the fixed effects, covariates and random effects included in the final mixed animal models**

| **Traits^1^** | **Fixed effects and covariates^2^** | **Random effects^3^** | |
| --- | --- | --- | --- |
|  |  | **Tested models** | **AIC**^4^ |
| TNB | FP^*^, CG_R^*^, fAge^*^, fAge2^*^ | y = fixed effects + $a$ + $pe$ + $ce$ + e | **952,176.8386** |
|  |  | y = fixed effects + $a$ + $pe$ + e | 954,991.6688 |
| NBA | FP^*^, CG_R^*^, fAge^*^, fAge2^*^ | y = fixed effects + $a$ + $pe$ + $ce$ + e | **936,948.8342** |
|  |  | y = fixed effects + $a$ + $pe$ + e | 939,354.9387 |
| NW | FP^*^, CG_R^*^, fAge^*^, fAge2^ns^, weanAge^*^, weanAge2^*^ | y = fixed effects + $a$ + $pe$ + $ce$ + e | **144,047.0015** |
|  |  | y = fixed effects + $a$ + $pe$ + e | 148,366.2718 |
| WW | Sex^*^, BP^*^, CG_G^*^, wAge^*^, weanAge2^ns^ | y = fixed effects + $a$ + $ce$ + e | **144,047.0014** |
|  |  | y = fixed effects + $a$ + e | 148,366.2718 |
| OW | Sex^*^, BP^*^, CG_G^*^, oAge^*^, oAge2^*^, wAge^*^, wAge2^*^ | y = fixed effects + $a$ + $ce$ + e | **952,059.7706** |
|  |  | y = fixed effects + $a$ + e | 956,683.7139 |
| MD | Sex^*^, BP^*^, CG_G^*^, oAge^*^, oAge2^*^, wAge^*^, wAge2^*^ | y = fixed effects + $a$ + $ce$ + e | **-2,205.0028** |
|  |  | y = fixed effects + $a$ + e | -1,512.6205 |
| BF | Sex^*^, BP^*^, CG_G^*^, oAge^*^, oAge2^*^, wAge^*^, wAge2^ns^ | y = fixed effects + $a$ + $ce$ + e | **952,059.7706** |
|  |  | y = fixed effects + $a$ + e | 956,683.7139 |

^1^ TNB: total number of piglets born; NBA: number of piglets born alive; NW: number of piglets weaned; WW: weaning weight (Kg); OW: off-test weight (Kg); MD: ultrasound muscle depth (mm); BF: ultrasound backfat thickness (mm).

^2^ FP: farrowing parity; BP: birth parity; CG_R: reproduction contemporary group; CG_G: growth contemporary group. fAge: linear effect of farrowing age; fAge2: quadratic effect of farrowing age; wAge: linear effect of weaning age; wAge2: quadratic effect of weaning age; oAge: linear effect of off-test age; oAge2: quadratic effect of off-test age. ^*^, *P* < 0.001; ^ns^, *P* > 0.1.

^3^ $a$: animal (additive genetic) effect; $pe$: animal permanent environmental effect across parities; $ce$: litter effect; AIC: Akaike information criterion.

^4^ The models with lower AIC values were selected and denoted in bold.

**Table S3 Standard errors of variance components estimates based on the homogenous (RNM1) and heterogeneous (RNM2) residuals**

| **Traits**^1^ | **RNM1**^2^ | | | | | | **RNM2** | | | | | | $\boldsymbol{d}_{\boldsymbol{0}}$**/** $\boldsymbol{d}_{\boldsymbol{1}}$^4^ |
| --- | --- | --- | --- | --- | --- | --- | --- | --- | --- | --- | --- | --- | --- |
|  | $a$ | | $pe$ | | $ce$ | | $a$ | | $pe$ | | $ce$ | |  |
| TNB^3^ | 0.0607 | 0.0273 | 0.0448 | 0.0193 | 0.0253 | 0.0089 | 0.0585 | 0.0236 | 0.0440 | 0.0109 | 0.0244 | 0.0054 | 2.1881 / 0.1796 |
|  | 0.0273 | 0.0193 | 0.0193 | 0.0105 | 0.0089 | 0.0036 | 0.0236 | 0.0191 | 0.0109 | 0.0028 | 0.0054 | 0.0013 |  |
| NBA | 0.0502 | 0.0282 | 0.0436 | 0.0249 | 0.0256 | 0.0154 | 0.0501 | 0.0284 | 0.0434 | 0.0261 | 0.0253 | 0.0156 | 2.0872 / 0.1287 |
|  | 0.0282 | 0.0292 | 0.0249 | 0.0448 | 0.0154 | 0.0306 | 0.0284 | 0.0297 | 0.0261 | 0.0447 | 0.0156 | 0.0308 |  |
| NW | 0.0651 | 0.0353 | 0.1126 | 0.0722 | 0.0847 | 0.0550 | 0.0651 | 0.0300 | 0.1114 | 0.0764 | 0.0839 | 0.0527 | 1.7774 / -0.0356 |
|  | 0.0353 | 0.0089 | 0.0722 | 0.0724 | 0.0550 | 0.0433 | 0.0300 | 0.0074 | 0.0764 | 0.0704 | 0.0527 | 0.0374 |  |
| WW | 0.0438 | 0.0230 | - | - | 0.0433 | 0.0192 | 0.0383 | 0.0205 | - | - | 0.0412 | 0.0168 | 0.6380 / 0.0540 |
|  | 0.0230 | 0.0271 | - | - | 0.0192 | 0.0003 | 0.0205 | 0.0180 | - | - | 0.0168 | 0.0002 |  |
| OW | 2.8212 | 1.0402 | - | - | 0.7898 | 0.3441 | 2.9714 | 1.1947 | - | - | 0.6957 | 0.0830 | 4.6375 / 0.2400 |
|  | 1.0402 | 0.7168 | - | - | 0.3441 | 0.0153 | 1.1947 | 0.4975 | - | - | 0.0830 | 0.0015 |  |
| MD | 0.9651 | 0.2068 | - | - | 0.2711 | 0.1340 | 0.9119 | 0.0862 | - | - | 0.2127 | 0.0744 | 3.1972 / -0.0428 |
|  | 0.2068 | 0.0188 | - | - | 0.1340 | 0.0853 | 0.0862 | 0.0094 | - | - | 0.0744 | 0.0359 |  |
| BF | 0.5257 | 0.1956 | - | - | 0.1196 | 0.0492 | 0.5226 | 0.1898 | - | - | 0.0883 | 0.0236 | 2.1454 / 0.0017 |
|  | 0.1956 | 0.1259 | - | - | 0.0492 | 0.0186 | 0.1898 | 0.0454 | - | - | 0.0236 | 0.0079 |  |

^1^ TNB: total number of piglets born; NBA: number of piglets born alive; NW: number of piglets weaned; WW: weaning weight (Kg); OW: off-test weight (Kg); MD: ultrasound muscle depth (mm); BF: ultrasound backfat thickness (mm).

^2^ $a$: animal (additive genetic) effect; $pe$: animal permanent environmental effect across parities; $ce$: litter effect.

^3^ In the 2×2 block between every trait and random effect, the diagonals, upper triangular and lower triangular represent the standard errors for variance, covariance and correlation for the intercept and slope coefficients, respectively.

^4^ $d_{0}$/$d_{1}$: the two coefficients for fitting the heterogeneous residuals under RNM2.

**Table S4 Spearman’s rank correlation of genomic estimated breeding values across three representative environmental gradients**

| **Trait** | **N of animals**^1^ | **15% *vs.* 50%**^2^ | | **50% *vs.* 85%** | | **15% *vs.* 85%** | |
| --- | --- | --- | --- | --- | --- | --- | --- |
|  |  | **Estimate** | **95% CI** | **Estimate** | **95% CI** | **Estimate** | **95% CI** |
| TNB | 1,630 | 0.6143 | 0.5823 - 0.6471 | 0.9239 | 0.9152 - 0.9331 | 0.2981 | 0.254 - 0.3434 |
| NBA | 1,618 | 0.6685 | 0.6391 - 0.6986 | 0.9037 | 0.8931 - 0.9153 | 0.3185 | 0.273 - 0.3631 |
| NW | 398 | 1.0000 | 1.0000 - 1.0000 | 1.0000 | 1.0000 - 1.0000 | 1.0000 | 1.0000 - 1.0000 |
| WW | 1,064 | 0.9969 | 0.9965 - 0.9976 | 0.9589 | 0.9535 - 0.9651 | 0.9344 | 0.9256 - 0.9438 |
| OW | 2,873 | 0.9995 | 0.9995 - 0.9996 | 0.9998 | 0.9998 - 0.9998 | 0.9988 | 0.9987 - 0.9989 |
| MD | 1,113 | 1.0000 | 1.0000 - 1.0000 | 1.0000 | 1.0000 - 1.0000 | 1.0000 | 1.0000 - 1.0000 |
| BF | 1,077 | 0.9980 | 0.9978 - 0.9984 | 0.9987 | 0.9986 - 0.9991 | 0.9938 | 0.993 - 0.9949 |

^1^ The rank correlation was calculated for animals that have more than 10 offspring and the accuracy of EBVs higher than 0.3.

^2^ Three representative environmental conditions were selected and compared, including the worse (15% quantile of environmental gradient), moderate (50% quantile), and better (85% quantile) environments. CI: confidence interval, which was calculated using bootstrap method (bootstrapped with 5000 times).

**Table S5 Genomic windows with the significantly explained variances and associated candidate genes for the four traits without G×E interaction**

| **Traits**^1^ | **Genomic windows**^2^ | | **Variance (%)**^3^ | | **Candidate genes** |
| --- | --- | --- | --- | --- | --- |
|  | **Chr** | **Positions (bp)** | **Int** | **Slo** |  |
| NW | SSC10 | 24,279,855-24,457,455 | *0.68* | *0.68* | *GPR37L1*, *ARL8A*, *PTPN7*, ENSSSCG00000049544, *LGR6*, ENSSSCG00000037298, *UBE2T*, *PPP1R12B* |
|  |  | 24,293,209-24,475,590 | *0.85* | *0.85* |  |
|  |  | 24,402,167-24,519,940 | *0.72* | *0.72* |  |
|  |  | 24,457,455-24,542,239 | *0.71* | *0.71* |  |
|  | SSCX | 41,908,692-41,875,471 | *0.58* | *0.58* | None |
| OW | SSC1 | 264,949,263-265,069,259 | *0.50* | *0.48* | None |
|  | SSC13 | 19,334,390-19,471,344 | *0.52* | *0.56* | *CLASP2* |
|  |  | 19,371,570-19,514,505 | *0.50* | *0.55* |  |
|  | SSC14 | 99,482,409-99,736,605 | *0.53* | *0.50* | ENSSSCG00000045184*, *MINPP1*, *PAPSS2* |
| MD | SSC7 | 17,707,473-17,835,129 | *0.63* | *0.62* | ENSSSCG00000046828*, ENSSSCG00000048107*, ENSSSCG00000050980* |
|  | SSC11 | 11,688,818-11,882,898 | *0.57* | *0.57* | *NBEA*, *DCLK1* |
|  | SSC12 | 56,161,319-56,214,880 | *0.55* | *0.55* | *DNAH9* |
|  |  | 56,141,634-56,240,724 | *0.96* | *0.95* |  |
|  |  | 56,124,653-56,285,163 | *0.69* | *0.69* |  |
|  |  | 56,197,640-56,304,422 | *0.81* | *0.80* |  |
|  |  | 56,214,880-56,324,680 | *0.74* | *0.73* |  |
|  | SSC14 | 134,570,084-134,625,180 | *0.59* | *0.59* | None |
|  | SSC16 | 30,711,806-30,827,133 | *0.50* | *0.50* | None |
|  |  | 30,756,112-30,879,331 | *0.51* | *0.51* |  |
| BF | SSC1 | 52,110,037-52,241,222 | *0.56* | 0.46 | *RIMS1* |
|  | SSC6 | 40,301,935-40,327,404 | *0.89* | *0.91* | *ZNF536* |
|  |  | 40,449,359-40,558,794 | *0.88* | *0.89* |  |
|  |  | 40,410,621-40,491,077 | *0.71* | *0.71* |  |

^1^ NW: number of piglets weaned; OW: off-test weight (Kg); MD: ultrasound muscle depth (mm); BF: ultrasound backfat thickness (mm).

^2^ Genomic windows are defined by the five adjacent SNPs that explained 0.5% or more of the total additive genetic variance. Chr: chromosomes. The positions are referred to Sscrofa11.1.

^3^ The explained genetic variances in percent for intercept (Int) and slope (Slo) by five adjacent SNPs. The explained variances with ≥ 0.5% are denoted in italics.

^4^ Candidate genes are represented by the gene symbol when available, otherwise by the Ensembl gene ID. The long noncoding RNA genes are marked by marked by an asterisk (*).

**Table S6 Related quantitative trait loci (QTLs) within relevant genomic regions for the four traits without G×E interaction**

| **Traits**^1^ | **Genomic regions (bp)**^2^ | **The related QTLs (number of reports)** |
| --- | --- | --- |
| NW | SSC10: 24,279,855 - 24,542,239 | Teat number (1), Melanoma susceptibility (1), Osteochondrosis score (1), Immunity response (2) |
|  | SSCX: 41,908,692 - 41,875,471 | Immunity response (1) |
| OW | SSC1: 264,949,263 - 265,069,259 | Average daily gain (9), Carcass length (1), Age at puberty (1), Body weight at birth (2), Body weight at weaning (2), Dressing percentage (1), Carcass weight (1) |
|  | SSC13: 19,334,390 - 19,514,505 | Average daily gain (5), Body weight at weaning (1), Age at puberty (1) |
|  | SSC14: 99,482,409 - 99,736,605 | Body weight (2), Carcass weight (2), Ham weight (3), Loin weight (1), Shoulder meat weight (1) |
| MD | SSC7: 17,707,473 - 17,835,129 | Loin muscle area (4), Loin muscle depth (2), Muscle moisture percentage (1), Meat color (1) |
|  | SSC11: 11,688,818 - 11,882,898 | Loin muscle area (1) |
|  | SSC12: 56,161,319 - 56,324,680 | Loin muscle area (3), Loin muscle depth (1), Meat color (4) |
|  | SSC14: 134,570,084 - 134,625,180 | Loin muscle area (3), Loin muscle depth (1), Meat color (3), Shear force (5) |
|  | SSC16: 30,711,806 - 30,879,331 | Loin muscle area (2), Meat color (4), Cooking loss (3), Shear force (2) |
| BF | SSC1: 52,110,037 - 52,241,222 | Backfat at rump (1), Loin fat percentage (1), Leaf fat weight (2), Backfat at rib (6), Fat thickness (6), backfat above muscle dorsi (1), Marbling (2), Muscle fat content (1), Subcutanous fat area (1), External fat on ham (1), Shoulder external fat weight (1), Fat to meat ratio (1) |
|  | SSC6: 40,301,935 - 40,491,077 | Intramuscular fat content (4), Average backfat thickness (5), Backfat at ribs (7), Average backfat thickness (2), Loin fat percentage (1), External fat on ham (1), Abdominal fat weight (1), Backfat weight (2), Shoulder external fat weight (1) |

^1^ NW: number of piglets weaned; OW: off-test weight (Kg); MD: ultrasound muscle depth (mm); BF: ultrasound backfat thickness (mm).

^2^ The genomic regions are concatenated by the overlapped genomic windows shown in Table 3, and the positions are referred to Sscrofa11.1.

**Table S7 Accuracies and reliabilities of genomic estimated breeding values for all animals**

| **Traits^1^** | **Items** | **Average accuracies (95% confidence interval)** |
| --- | --- | --- |
| TNB | Intercept | 0.5935 (0.5928-0.5942) |
|  | Slope | 0.4632 (0.4626-0.4639) |
| NBA | Intercept | 0.5891 (0.5884-0.5898) |
|  | Slope | 0.4236 (0.423-0.4242) |
| NW | Intercept | 0.3926 (0.3905-0.3947) |
|  | Slope | 0.3898 (0.3877-0.392) |
| WW | Intercept | 0.4607 (0.4599-0.4616) |
|  | Slope | 0.2918 (0.2911-0.2925) |
| OW | Intercept | 0.6487 (0.6481-0.6493) |
|  | Slope | 0.6059 (0.6053-0.6064) |
| MD | Intercept | 0.616 (0.6142-0.6177) |
|  | Slope | 0.58 (0.5783-0.5816) |
| BF | Intercept | 0.5935 (0.5928-0.5942) |
|  | Slope | 0.4632 (0.4626-0.4639) |

^1^ TNB: total number of piglets born; NBA: number of piglets born alive; NW: number of piglets weaned; WW: weaning weight (Kg); OW: off-test weight (Kg); MD: ultrasound muscle depth (mm); BF: ultrasound backfat thickness (mm).

**Table S8 Sample sizes and accuracies used for calculating the approximate genetic correlations (weighted Pearson coefficients)**

| **Item 1** | **Item 2** | **N of animals** | **Average accuracies of GEBVs (95% confidence interval)** | |
| --- | --- | --- | --- | --- |
|  |  |  | **Item 1** | **Item 2** |
| TNB_Itc | NBA_Itc | 16,019 | 0.7221 (0.7212-0.723) | 0.7102 (0.7092-0.7112) |
| TNB_Itc | NBA_Slp | 16,019 | 0.7221 (0.7212-0.723) | 0.5412 (0.5405-0.542) |
| TNB_Slp | NBA_Itc | 16,019 | 0.5947 (0.594-0.5953) | 0.7102 (0.7092-0.7112) |
| TNB_Slp | NBA_Slp | 16,019 | 0.5947 (0.594-0.5953) | 0.5412 (0.5405-0.542) |
| TNB_Itc | NW_Itc | 7,710 | 0.7699 (0.769-0.7709) | 0.4885 (0.487-0.49) |
| TNB_Itc | NW_Slp | 7,710 | 0.7699 (0.769-0.7709) | 0.4855 (0.484-0.487) |
| TNB_Slp | NW_Itc | 7,710 | 0.6135 (0.6124-0.6145) | 0.4885 (0.487-0.49) |
| TNB_Slp | NW_Slp | 7,710 | 0.6135 (0.6124-0.6145) | 0.4855 (0.484-0.487) |
| TNB_Itc | WW_Itc | 2,252 | 0.7713 (0.7698-0.7729) | 0.5662 (0.5642-0.5681) |
| TNB_Itc | WW_Slp | 2,252 | 0.7713 (0.7698-0.7729) | 0.3911 (0.3897-0.3924) |
| TNB_Slp | WW_Itc | 2,252 | 0.6181 (0.6163-0.6199) | 0.5662 (0.5642-0.5681) |
| TNB_Slp | WW_Slp | 2,252 | 0.6181 (0.6163-0.6199) | 0.3911 (0.3897-0.3924) |
| TNB_Itc | OW_Itc | 9,521 | 0.7533 (0.7522-0.7544) | 0.7919 (0.7908-0.793) |
| TNB_Itc | OW_Slp | 9,521 | 0.7533 (0.7522-0.7544) | 0.7475 (0.7464-0.7485) |
| TNB_Slp | OW_Itc | 9,521 | 0.6097 (0.6088-0.6106) | 0.7919 (0.7908-0.793) |
| TNB_Slp | OW_Slp | 9,521 | 0.6097 (0.6088-0.6106) | 0.7475 (0.7464-0.7485) |
| TNB_Itc | MD_Itc | 4,210 | 0.7737 (0.7725-0.7748) | 0.7633 (0.762-0.7647) |
| TNB_Itc | MD_Slp | 4,210 | 0.7737 (0.7725-0.7748) | 0.7207 (0.7194-0.7219) |
| TNB_Slp | MD_Itc | 4,210 | 0.6136 (0.6121-0.615) | 0.7633 (0.762-0.7647) |
| TNB_Slp | MD_Slp | 4,210 | 0.6136 (0.6121-0.615) | 0.7207 (0.7194-0.7219) |
| TNB_Itc | BF_Itc | 5,596 | 0.7725 (0.7715-0.7736) | 0.7798 (0.7782-0.7814) |
| TNB_Itc | BF_Slp | 5,596 | 0.7725 (0.7715-0.7736) | 0.4582 (0.4572-0.4592) |
| TNB_Slp | BF_Itc | 5,596 | 0.6161 (0.6149-0.6173) | 0.7798 (0.7782-0.7814) |
| TNB_Slp | BF_Slp | 5,596 | 0.6161 (0.6149-0.6173) | 0.4582 (0.4572-0.4592) |
| NBA_Itc | NW_Itc | 9,700 | 0.7469 (0.7457-0.748) | 0.4766 (0.4752-0.4779) |
| NBA_Itc | NW_Slp | 9,700 | 0.7469 (0.7457-0.748) | 0.4735 (0.4722-0.4749) |
| NBA_Slp | NW_Itc | 9,700 | 0.5516 (0.5505-0.5527) | 0.4766 (0.4752-0.4779) |
| NBA_Slp | NW_Slp | 9,700 | 0.5516 (0.5505-0.5527) | 0.4735 (0.4722-0.4749) |
| NBA_Itc | WW_Itc | 2,391 | 0.7628 (0.7609-0.7646) | 0.5625 (0.5605-0.5645) |
| NBA_Itc | WW_Slp | 2,391 | 0.7628 (0.7609-0.7646) | 0.39 (0.3887-0.3912) |
| NBA_Slp | WW_Itc | 2,391 | 0.5721 (0.5702-0.5741) | 0.5625 (0.5605-0.5645) |
| NBA_Slp | WW_Slp | 2,391 | 0.5721 (0.5702-0.5741) | 0.39 (0.3887-0.3912) |
| NBA_Itc | OW_Itc | 11,601 | 0.7336 (0.7324-0.7347) | 0.7835 (0.7825-0.7845) |
| NBA_Itc | OW_Slp | 11,601 | 0.7336 (0.7324-0.7347) | 0.7392 (0.7382-0.7401) |
| NBA_Slp | OW_Itc | 11,601 | 0.5455 (0.5445-0.5465) | 0.7835 (0.7825-0.7845) |
| NBA_Slp | OW_Slp | 11,601 | 0.5455 (0.5445-0.5465) | 0.7392 (0.7382-0.7401) |
| NBA_Itc | MD_Itc | 4,745 | 0.763 (0.7618-0.7642) | 0.762 (0.7607-0.7632) |
| NBA_Itc | MD_Slp | 4,745 | 0.763 (0.7618-0.7642) | 0.719 (0.7178-0.7201) |
| NBA_Slp | MD_Itc | 4,745 | 0.5607 (0.5592-0.5623) | 0.762 (0.7607-0.7632) |
| NBA_Slp | MD_Slp | 4,745 | 0.5607 (0.5592-0.5623) | 0.719 (0.7178-0.7201) |
| NBA_Itc | BF_Itc | 6,144 | 0.7613 (0.7601-0.7624) | 0.7769 (0.7753-0.7785) |
| NBA_Itc | BF_Slp | 6,144 | 0.7613 (0.7601-0.7624) | 0.4558 (0.4549-0.4568) |
| NBA_Slp | BF_Itc | 6,144 | 0.5653 (0.564-0.5666) | 0.7769 (0.7753-0.7785) |
| NBA_Slp | BF_Slp | 6,144 | 0.5653 (0.564-0.5666) | 0.4558 (0.4549-0.4568) |
| NW_Itc | WW_Itc | 2,392 | 0.5351 (0.5331-0.5372) | 0.5623 (0.5603-0.5643) |
| NW_Itc | WW_Slp | 2,392 | 0.5351 (0.5331-0.5372) | 0.39 (0.3887-0.3912) |
| NW_Slp | WW_Itc | 2,392 | 0.5321 (0.5301-0.5342) | 0.5623 (0.5603-0.5643) |
| NW_Slp | WW_Slp | 2,392 | 0.5321 (0.5301-0.5342) | 0.39 (0.3887-0.3912) |
| NW_Itc | OW_Itc | 8,810 | 0.4832 (0.4819-0.4846) | 0.8062 (0.8053-0.8071) |
| NW_Itc | OW_Slp | 8,810 | 0.4832 (0.4819-0.4846) | 0.7612 (0.7603-0.7621) |
| NW_Slp | OW_Itc | 8,810 | 0.4801 (0.4787-0.4815) | 0.8062 (0.8053-0.8071) |
| NW_Slp | OW_Slp | 8,810 | 0.4801 (0.4787-0.4815) | 0.7612 (0.7603-0.7621) |
| NW_Itc | MD_Itc | 4,856 | 0.5224 (0.5209-0.5239) | 0.7617 (0.7605-0.7629) |
| NW_Itc | MD_Slp | 4,856 | 0.5224 (0.5209-0.5239) | 0.7186 (0.7174-0.7197) |
| NW_Slp | MD_Itc | 4,856 | 0.519 (0.5175-0.5205) | 0.7617 (0.7605-0.7629) |
| NW_Slp | MD_Slp | 4,856 | 0.519 (0.5175-0.5205) | 0.7186 (0.7174-0.7197) |
| NW_Itc | BF_Itc | 6,208 | 0.5104 (0.509-0.5117) | 0.7768 (0.7752-0.7784) |
| NW_Itc | BF_Slp | 6,208 | 0.5104 (0.509-0.5117) | 0.4556 (0.4547-0.4565) |
| NW_Slp | BF_Itc | 6,208 | 0.5073 (0.5059-0.5086) | 0.7768 (0.7752-0.7784) |
| NW_Slp | BF_Slp | 6,208 | 0.5073 (0.5059-0.5086) | 0.4556 (0.4547-0.4565) |
| WW_Itc | OW_Itc | 4,140 | 0.5423 (0.5409-0.5437) | 0.7826 (0.7807-0.7845) |
| WW_Itc | OW_Slp | 4,140 | 0.5423 (0.5409-0.5437) | 0.742 (0.7402-0.7438) |
| WW_Slp | OW_Itc | 4,140 | 0.3792 (0.3784-0.3801) | 0.7826 (0.7807-0.7845) |
| WW_Slp | OW_Slp | 4,140 | 0.3792 (0.3784-0.3801) | 0.742 (0.7402-0.7438) |
| WW_Itc | MD_Itc | 3,554 | 0.5502 (0.5488-0.5517) | 0.7465 (0.7447-0.7483) |
| WW_Itc | MD_Slp | 3,554 | 0.5502 (0.5488-0.5517) | 0.7069 (0.7052-0.7086) |
| WW_Slp | MD_Itc | 3,554 | 0.3816 (0.3807-0.3825) | 0.7465 (0.7447-0.7483) |
| WW_Slp | MD_Slp | 3,554 | 0.3816 (0.3807-0.3825) | 0.7069 (0.7052-0.7086) |
| WW_Itc | BF_Itc | 3,846 | 0.5437 (0.5423-0.5452) | 0.7767 (0.7747-0.7786) |
| WW_Itc | BF_Slp | 3,846 | 0.5437 (0.5423-0.5452) | 0.4555 (0.4542-0.4567) |
| WW_Slp | BF_Itc | 3,846 | 0.381 (0.3801-0.3819) | 0.7767 (0.7747-0.7786) |
| WW_Slp | BF_Slp | 3,846 | 0.381 (0.3801-0.3819) | 0.4555 (0.4542-0.4567) |
| OW_Itc | MD_Itc | 8,643 | 0.7799 (0.7787-0.7811) | 0.7317 (0.7307-0.7327) |
| OW_Itc | MD_Slp | 8,643 | 0.7799 (0.7787-0.7811) | 0.691 (0.6901-0.6919) |
| OW_Slp | MD_Itc | 8,643 | 0.7388 (0.7377-0.74) | 0.7317 (0.7307-0.7327) |
| OW_Slp | MD_Slp | 8,643 | 0.7388 (0.7377-0.74) | 0.691 (0.6901-0.6919) |
| OW_Itc | BF_Itc | 12,273 | 0.7647 (0.7636-0.7657) | 0.7574 (0.7566-0.7583) |
| OW_Itc | BF_Slp | 12,273 | 0.7647 (0.7636-0.7657) | 0.4383 (0.4377-0.4389) |
| OW_Slp | BF_Itc | 12,273 | 0.725 (0.724-0.7259) | 0.7574 (0.7566-0.7583) |
| OW_Slp | BF_Slp | 12,273 | 0.725 (0.724-0.7259) | 0.4383 (0.4377-0.4389) |
| MD_Itc | BF_Itc | 7,283 | 0.7356 (0.7345-0.7367) | 0.7813 (0.7802-0.7823) |
| MD_Itc | BF_Slp | 7,283 | 0.7356 (0.7345-0.7367) | 0.4506 (0.4498-0.4515) |
| MD_Slp | BF_Itc | 7,283 | 0.6952 (0.6941-0.6962) | 0.7813 (0.7802-0.7823) |
| MD_Slp | BF_Slp | 7,283 | 0.6952 (0.6941-0.6962) | 0.4506 (0.4498-0.4515) |

^1^ The Items are referred to the concatenation of traits and RNM items, for instances, “TNB_Itc” and “TNB_Slp” represent the RNM intercept (Itc) and slope (Slp) of the trait “TNB”, respectively. The intra-trait genetic correlation between RNM intercept and slope is the real value obtained during the previous estimation of variance components. TNB: total number of piglets born; NBA: number of piglets born alive; NW: number of piglets weaned; WW: weaning weight (Kg); OW: off-test weight (Kg); MD: ultrasound muscle depth (mm); BF: ultrasound backfat thickness (mm).

**Table S9 Detailed information for the candidate genes found in this study**

| **Ensembl gene ID** | **Gene symbol** | **Chr.** | **Start position (bp)** | **End position (bp)** | **Gene biotype** | **Chr. strand** | **Entrezgene description** |
| --- | --- | --- | --- | --- | --- | --- | --- |
| ENSSSCG00000007606 | *TRRAP* | 3 | 5,909,237 | 6,029,340 | protein_coding | 1 | transformation/transcription domain associated protein |
| ENSSSCG00000039473 | NA | 12 | 37,729,098 | 37,944,050 | protein_coding | 1 | ubiquitin specific peptidase 32 |
| ENSSSCG00000012110 | *MID1* | X | 7,237,942 | 7,620,011 | protein_coding | -1 | midline 1 |
| ENSSSCG00000012172 | *ACOT9* | X | 19,834,614 | 19,952,081 | protein_coding | -1 | acyl-CoA thioesterase 9 |
| ENSSSCG00000012173 | *SAT1* | X | 19,907,900 | 19,910,895 | protein_coding | 1 | spermidine/spermine N1-acetyltransferase 1 |
| ENSSSCG00000012174 | *APOO* | X | 19,947,188 | 20,017,727 | protein_coding | -1 | apolipoprotein O |
| ENSSSCG00000012175 | *CXorf58* | X | 20,021,113 | 20,051,343 | protein_coding | 1 | chromosome X CXorf58 homolog |
| ENSSSCG00000012587 | *TRPC5* | X | 91,721,617 | 91,988,120 | protein_coding | -1 | transient receptor potential cation channel subfamily C member 5 |
| ENSSSCG00000000769 | NA | 5 | 69,849,002 | 69,875,853 | protein_coding | -1 | BH3 interacting domain death agonist |
| ENSSSCG00000000770 | *MICAL3* | 5 | 69,887,884 | 70,133,277 | protein_coding | -1 | microtubule associated monooxygenase, calponin and LIM domain containing 3 |
| ENSSSCG00000012741 | *MAMLD1* | X | 122,120,201 | 122,234,476 | protein_coding | 1 | mastermind like domain containing 1 |
| ENSSSCG00000012742 | *MTM1* | X | 122,286,916 | 122,379,299 | protein_coding | 1 | myotubularin 1 |
| ENSSSCG00000038768 | *GPR37L1* | 10 | 24,307,057 | 24,315,104 | protein_coding | 1 | G protein-coupled receptor 37 like 1 |
| ENSSSCG00000036967 | *ARL8A* | 10 | 24,314,932 | 24,324,229 | protein_coding | -1 | ADP ribosylation factor like GTPase 8A |
| ENSSSCG00000037669 | *PTPN7* | 10 | 24,324,436 | 24,336,419 | protein_coding | -1 | protein tyrosine phosphatase non-receptor type 7 |
| ENSSSCG00000049544 | NA | 10 | 24,338,560 | 24,357,984 | protein_coding | 1 | receptor-type tyrosine-protein phosphatase V-like |
| ENSSSCG00000044583 | *LGR6* | 10 | 24,359,874 | 24,454,431 | protein_coding | 1 | leucine rich repeat containing G protein-coupled receptor 6 |
| ENSSSCG00000037298 | NA | 10 | 24,413,687 | 24,415,099 | protein_coding | 1 | NA |
| ENSSSCG00000010923 | *UBE2T* | 10 | 24,454,495 | 24,459,057 | protein_coding | -1 | ubiquitin conjugating enzyme E2 T |
| ENSSSCG00000010925 | *PPP1R12B* | 10 | 24,465,041 | 24,637,360 | protein_coding | 1 | protein phosphatase 1 regulatory subunit 12B |
| ENSSSCG00000004832 | *UBE3A* | 1 | 141,887,168 | 141,997,241 | protein_coding | 1 | ubiquitin protein ligase E3A |
| ENSSSCG00000050514 | NA | 1 | 142,034,421 | 142,082,626 | lncRNA | -1 | NA |
| ENSSSCG00000000528 | *PKP2* | 5 | 41,424,845 | 41,508,868 | protein_coding | 1 | plakophilin 2 |
| ENSSSCG00000027429 | *YARS2* | 5 | 41,561,626 | 41,584,818 | protein_coding | 1 | tyrosyl-tRNA synthetase 2 |
| ENSSSCG00000000530 | NA | 5 | 41,570,282 | 41,879,062 | protein_coding | -1 | FYVE, RhoGEF and PH domain containing 4 |
| ENSSSCG00000000767 | *ATP6V1E1* | 5 | 69,747,700 | 69,771,990 | protein_coding | -1 | ATPase H+ transporting V1 subunit E1 |
| ENSSSCG00000040373 | *BCL2L13* | 5 | 69,778,950 | 69,855,297 | protein_coding | 1 | BCL2 like 13 |
| ENSSSCG00000012585 | *DCX* | X | 91,323,768 | 91,644,567 | protein_coding | -1 | doublecortin |
| ENSSSCG00000011236 | *CLASP2* | 13 | 19,305,180 | 19,510,585 | protein_coding | -1 | cytoplasmic linker associated protein 2 |
| ENSSSCG00000045184 | NA | 14 | 99,458,908 | 99,574,388 | lncRNA | -1 | NA |
| ENSSSCG00000010435 | *MINPP1* | 14 | 99,574,440 | 99,628,175 | protein_coding | 1 | multiple inositol-polyphosphate phosphatase 1 |
| ENSSSCG00000010437 | *PAPSS2* | 14 | 99,685,578 | 99,808,389 | protein_coding | 1 | 3'-phosphoadenosine 5'-phosphosulfate synthase 2 |
| ENSSSCG00000046828 | NA | 7 | 17,768,054 | 17,774,533 | lncRNA | -1 | NA |
| ENSSSCG00000048107 | NA | 7 | 17,781,038 | 17,783,269 | lncRNA | 1 | NA |
| ENSSSCG00000050980 | NA | 7 | 17,782,321 | 17,787,585 | lncRNA | -1 | NA |
| ENSSSCG00000034348 | *NBEA* | 11 | 11,084,534 | 11,722,852 | protein_coding | 1 | neurobeachin |
| ENSSSCG00000033919 | *DCLK1* | 11 | 11,815,803 | 12,198,630 | protein_coding | -1 | doublecortin like kinase 1 |
| ENSSSCG00000018015 | *DNAH9* | 12 | 56,045,140 | 56,358,236 | protein_coding | 1 | dynein axonemal heavy chain 9 |
| ENSSSCG00000004280 | *RIMS1* | 1 | 51,785,495 | 52,244,934 | protein_coding | 1 | regulating synaptic membrane exocytosis 1 |
| ENSSSCG00000029234 | *ZNF536* | 6 | 40,292,351 | 40,771,730 | protein_coding | 1 | zinc finger protein 536 |

Chr: chromosome.
