# Supplementary material for: Genotype-by-environment interactions for reproduction, body composition, and growth traits in maternal-line pigs based on single-step genomic reaction norms
Source: Genet Sel Evol. 2021 Jun 17;53:51. doi: 10.1186/s12711-021-00645-y (PMC8212483; doi:10.1186/s12711-021-00645-y)
Supplement: Supplementary file 2 — Additional file 2: Fig. S1. Density distribution of the estimated effects of contemporary groups. Fig. S2. Genetic correlations across environmental gradients using the optimal reaction norm models for the four traits without G × E interaction. Fig. S3. Heritability estimates using reaction norm models with homogenous (RNM1) and heterogeneous (RNM2) residual variances for the four traits without G × E interaction. Fig. S4. Genomic estimated breeding values using the optimal reaction norm models for 20 sires with the highest and lowest reaction norm slopes, respectively (for the four traits without G × E interaction). Fig. S5. Miami plots for the proportion of explained variance by 5-SNP sliding genomic windows for the four traits without clear G × E interaction. Fig. S6. Biological processes involved for all candidate genes identified in this study. Fig. S7. Five enriched terms of Human Phenotype Ontology for the number of piglets born alive (NBA). Fig. S8. Heritability estimates for the total number of piglets born (TNB) and number of piglets born alive (NBA) across environmental gradients using three scenarios regarding the X-chromosome markers. [file 12711_2021_645_MOESM2_ESM.docx]

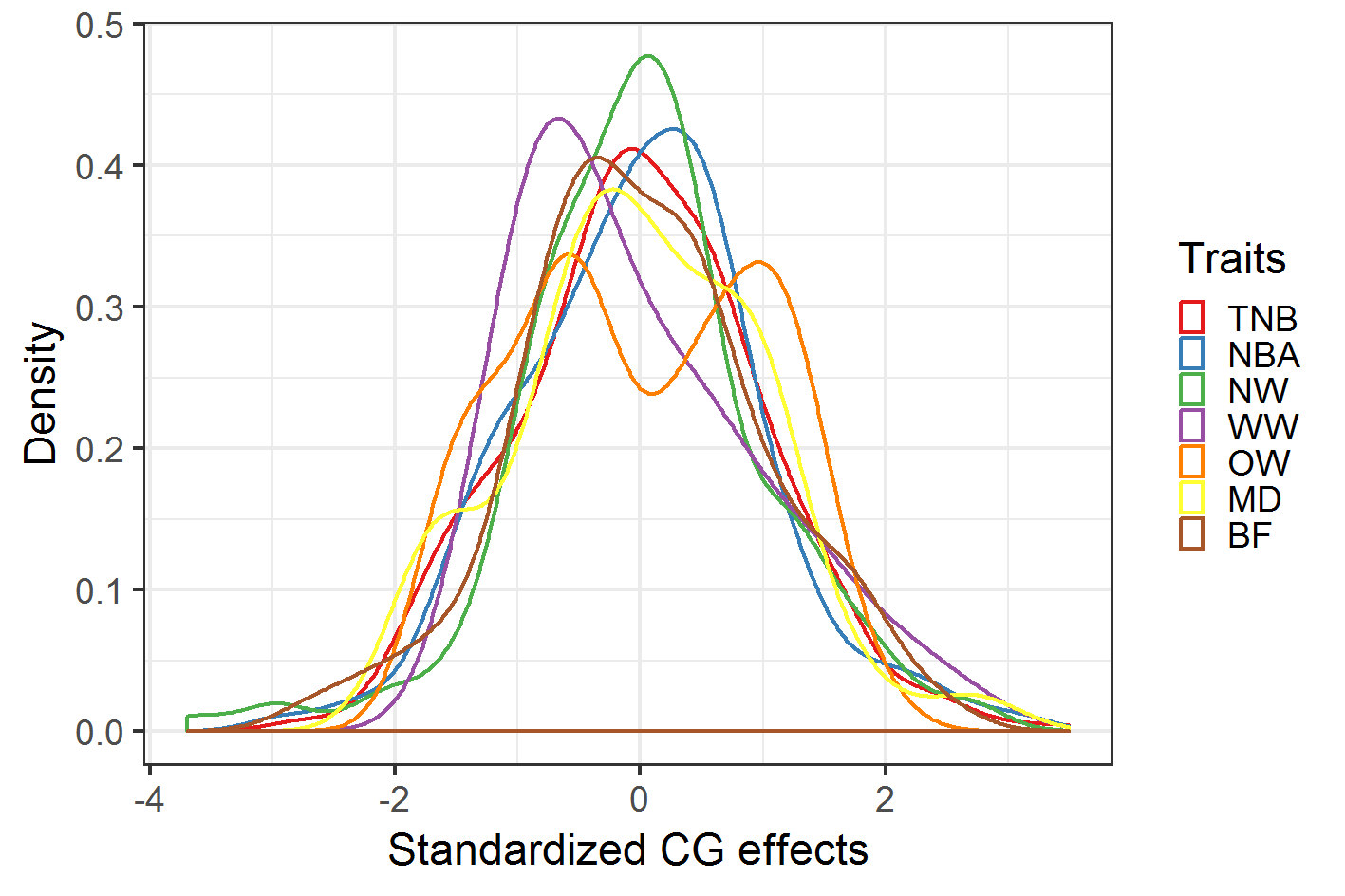


**Figure S1 Density distribution of the estimated effects of contemporary groups.** TNB: total number of piglets born; NBA: number of piglets born alive; NW: number of piglets weaned; WW: weaning weight (Kg); OW: off-test weight (Kg); MD: ultrasound muscle depth (mm); BF: ultrasound backfat thickness (mm).

**
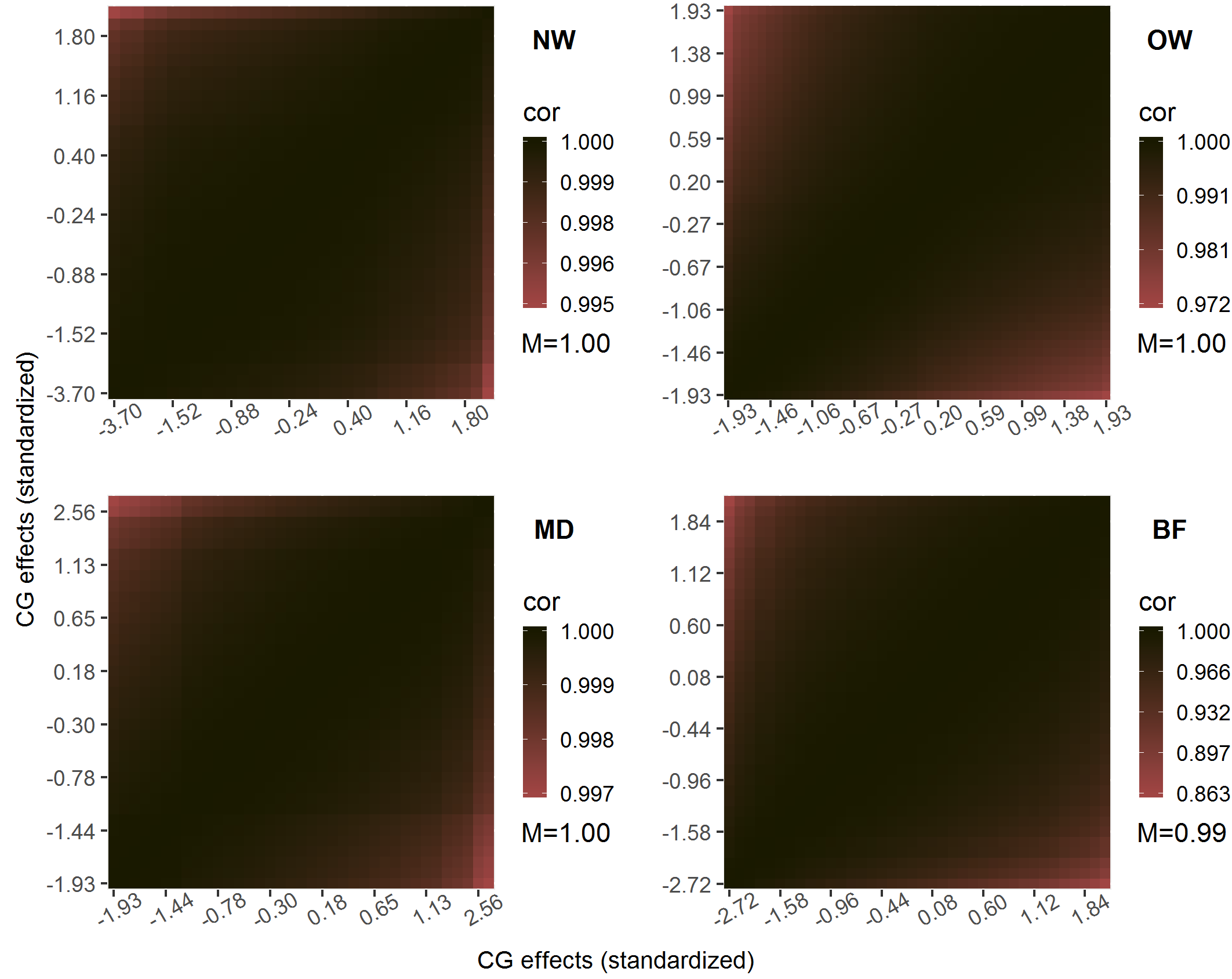
**

**Figure S2** **Genetic correlations across environmental gradients using the optimal reaction norm models for the four traits without G×E interaction**. The Pearson correlation coefficient (cor) are represented by colours with the mean values (M) shown below. NW: number of piglets weaned; OW: off-test weight (Kg); MD: ultrasound muscle depth (mm); BF: ultrasound backfat thickness (mm).


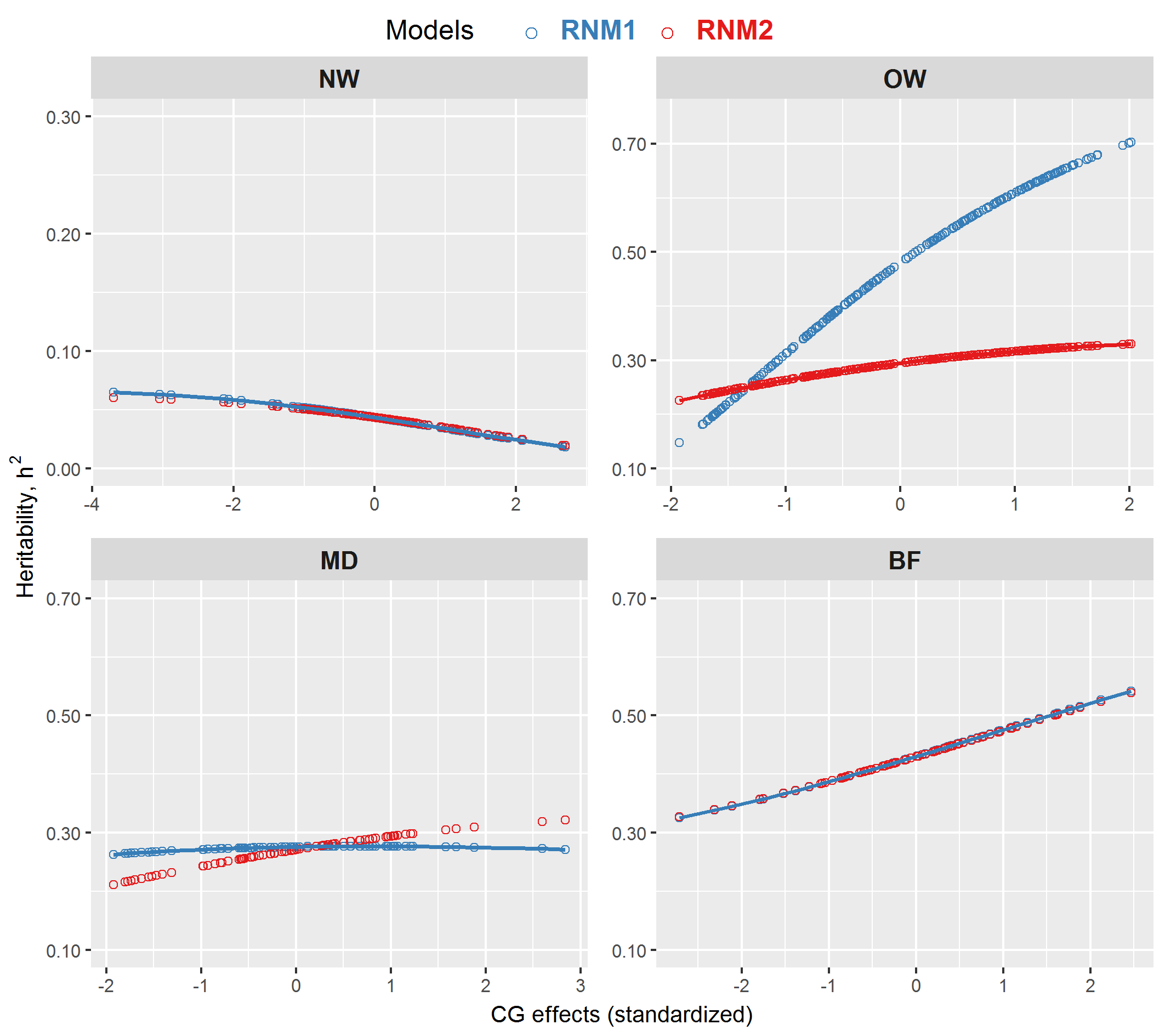


**Figure S3 Heritability estimates using reaction norm models with homogenous (RNM1) and heterogeneous (RNM2) residual variances for the four traits without G×E interaction**. The respective optimal RNMs for every trait were marked by the solid lines. NW: number of piglets weaned; OW: off-test weight (Kg); MD: ultrasound muscle depth (mm); BF: ultrasound backfat thickness (mm).


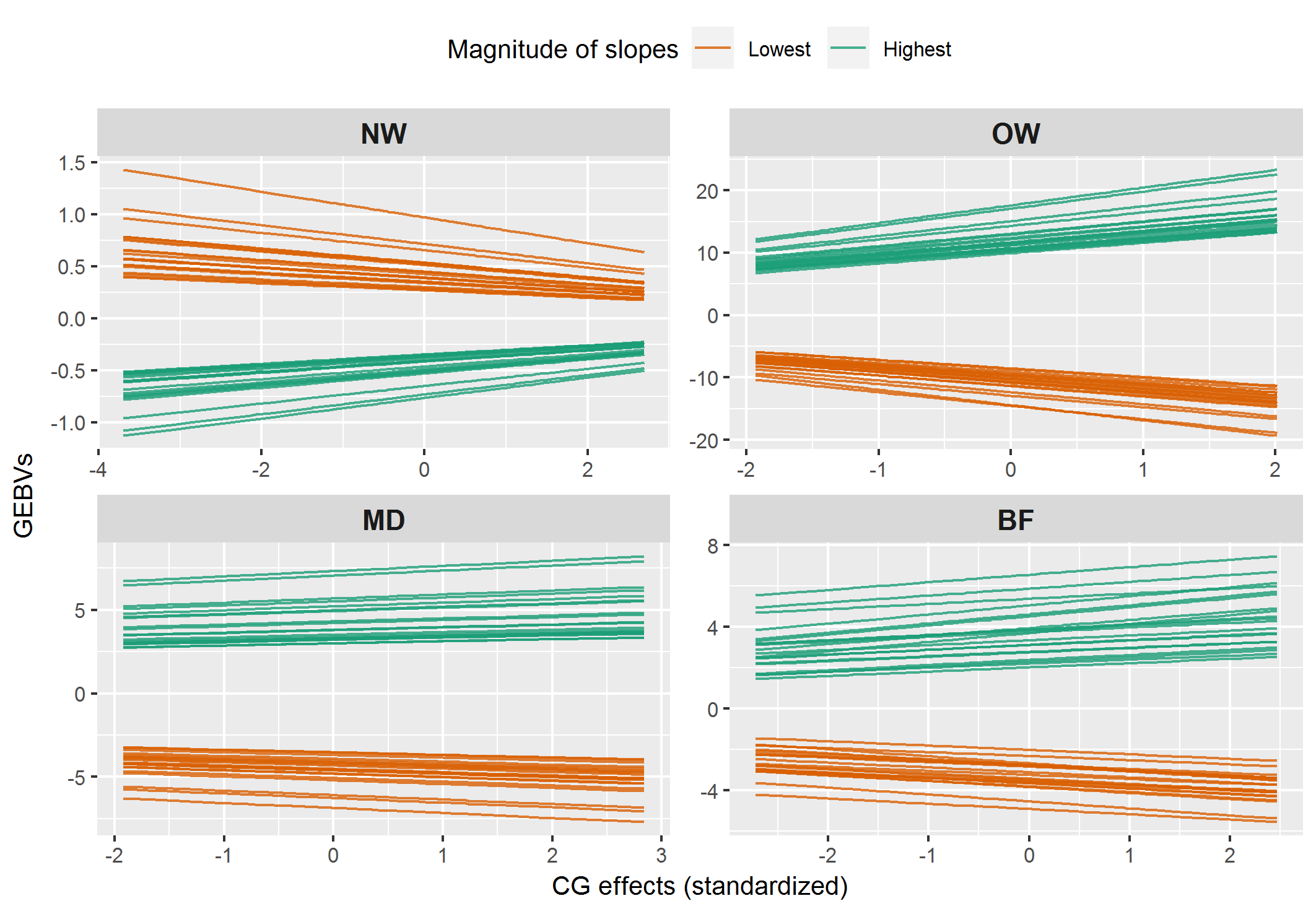


**Figure S4 Genomic estimated breeding values using the optimal reaction norm models for 20 sires with highest and lowest reaction norm slopes, respectively (for the four traits without G×E interaction)**. NW: number of piglets weaned; OW: off-test weight (Kg); MD: ultrasound muscle depth (mm); BF: ultrasound backfat thickness (mm).


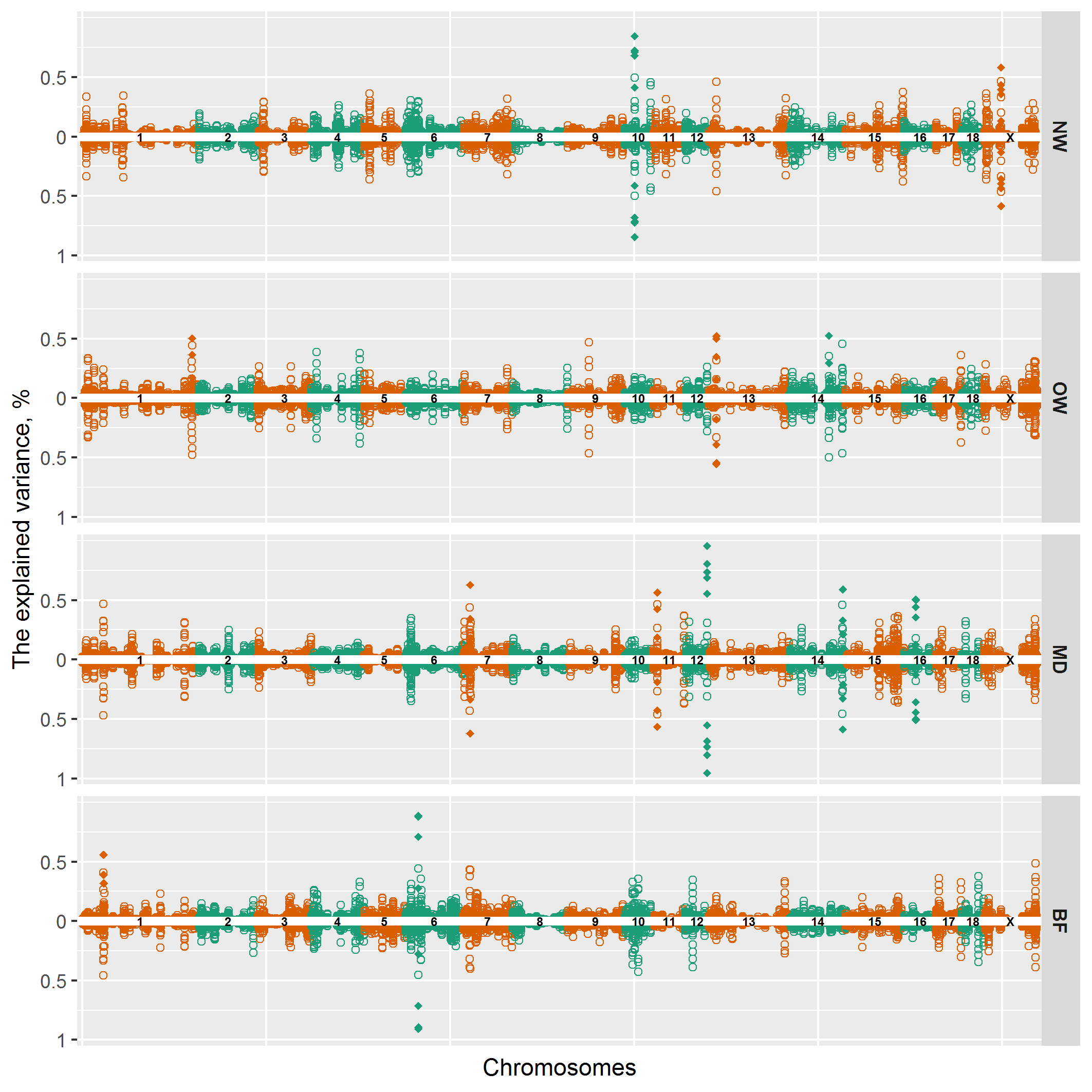


**Figure S5 Miami plots for the proportion of explained variance by 5-SNP moving genomic windows for the four traits without G×E interaction**. The intercept and slope terms of reaction norm model are placed on upper and bottom arms of y-axis, respectively. Each open dot represents a SNP, while all SNPs within the significant genomic windows are denoted as solid diamonds. NW: number of piglets weaned; OW: off-test weight (Kg); MD: ultrasound muscle depth (mm); BF: ultrasound backfat thickness (mm).

**
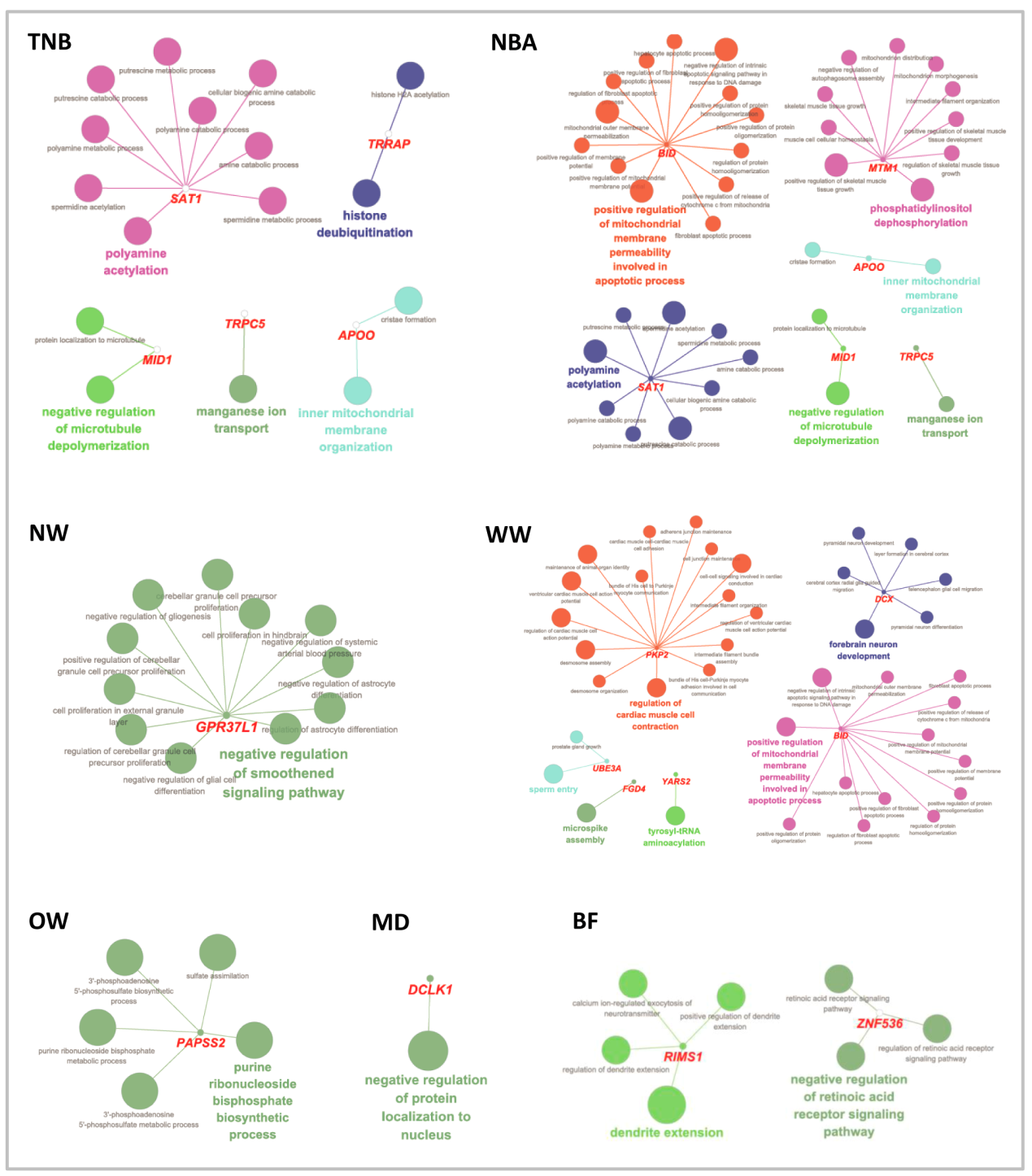
**

**Figure S6 Biological processes involved for all candidate genes**. Biological process shared at least one gene. Network specificity: little lower than medium; kappa score: 0.4. The gene symbols are shown in red. TNB: total number of piglets born; NBA: number of piglets born alive; NW: number of piglets weaned; WW: weaning weight (Kg); OW: off-test weight (Kg); MD: ultrasound muscle depth (mm); BF: ultrasound backfat thickness (mm).


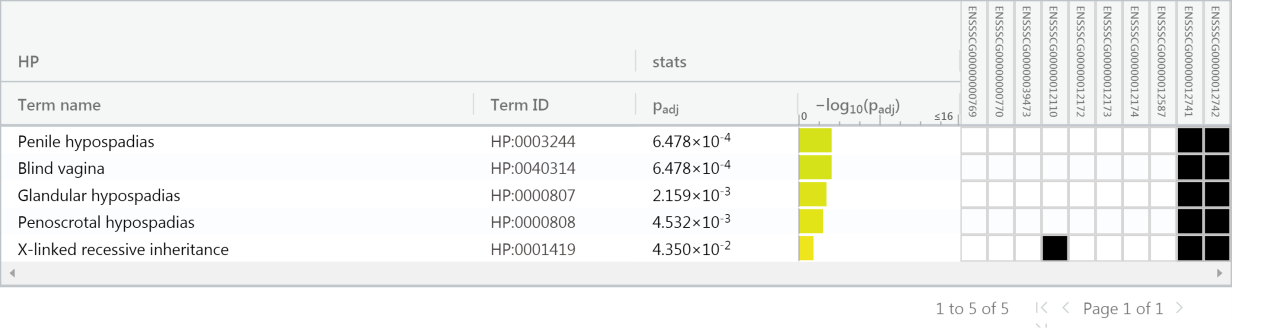
**Figure S7 Five enriched terms of Human Phenotype Ontology for number of piglets born alive (NBA)**. The detailed information for these genes is shown in Table S9.

**
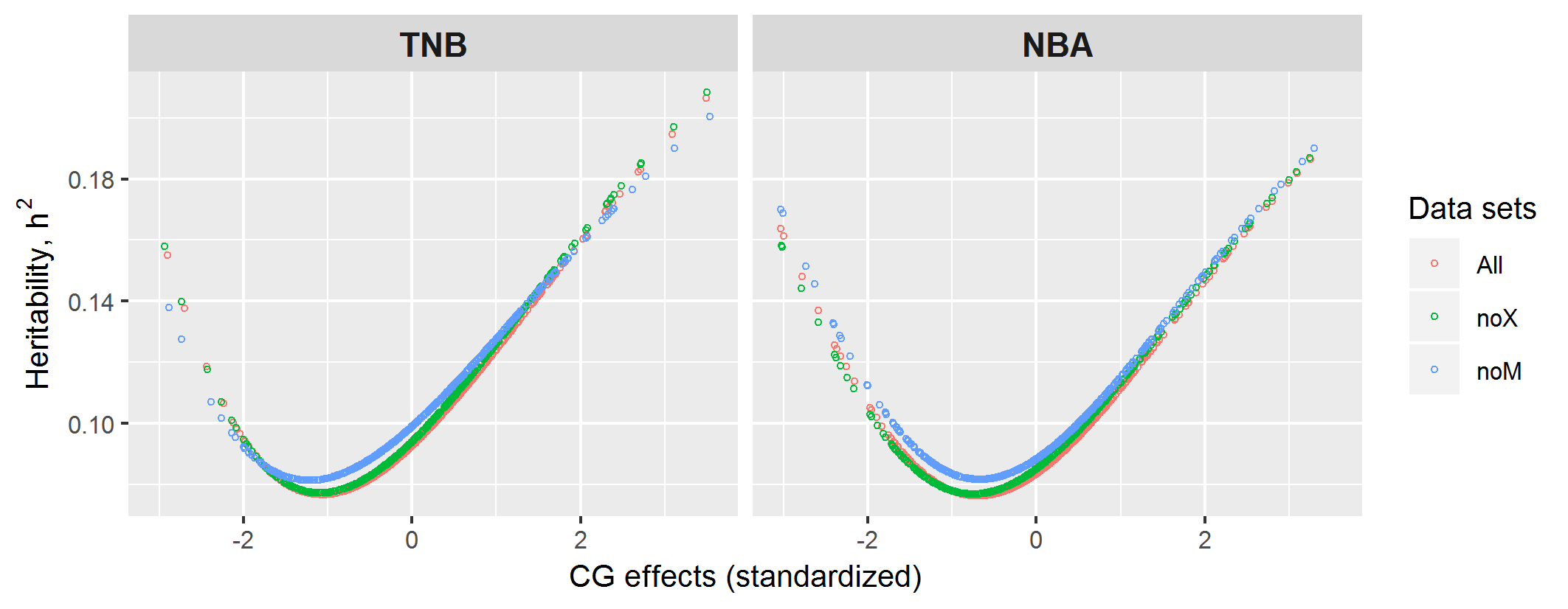
**

**Figure S8 Heritability estimates for total number of piglets born (TNB) and number of piglets born alive (NBA) across environmental gradients using three scenarios regarding the X-chromosome markers**. First, the X-chromosome markers were included for all animals (All), which is same to that in Figure 2. Second, we excluded the X-chromosome markers and only used the autosomes for all animals (noX). Third, we included the X-chromosome markers but removed the genotyped males (noM).
