# Supplementary material for: Genotype-by-environment interactions for reproduction, body composition, and growth traits in maternal-line pigs based on single-step genomic reaction norms
Source: Genet Sel Evol. 2021 Jun 17;53:51. doi: 10.1186/s12711-021-00645-y (PMC8212483; doi:10.1186/s12711-021-00645-y)
Supplement: Supplementary file 3 — Additional file 3: Detailed results for the four traits that did not show significant G × E interactions. [file 12711_2021_645_MOESM3_ESM.docx]

**Detailed results for the four traits that did not show significant G×E interactions**

**Heritabilities and breeding values across environments**

For the four traits that did not show significant G×E interaction (i.e., NW, OW, MD, and BF), we also estimated their genetic parameters [see Additional file 2 Figure S2 and S3]. Using either RNM1 or RNM2, similar heritabilities were estimated for both NW and BF; and their heritabilities decreased almost linearly (from 0.07 to 0.02) or increased (from 0.33 to 0.54) with respect to the worst and the best environmental conditions, respectively. The heritability was more stable over environmental gradient under RNM2 for OW, and under RNM1 for MD. However, both RNM1 and RNM2 yielded greater heritabilities with improved environmental conditions for OW.

In contrast to the three traits with significant G×E interaction, no re-ranking of GEBVs was observed for NW, OW, MD, and BF (see Additional file 2 Figure S4). However, the absolute values of GEBVs varied greatly among different environments for the traits of NW and OW, while they were kept approximately constant for MD and BF across environments.

**Genome-wide association studies and functional analyses**

Two, three, five, and two genomic regions (derived from 23 genomic windows) were significantly associated with NW, OW, MD, and BF, respectively (see Additional file 2 Figure S5, and Additional file 1 Table S5). Among them, one genomic window on SSC14 was shared between MD and TNB, and one on SSC5 between NBA and WW. However, no genomic region was shared among the four traits. Furthermore, the significant genomic windows exhibited very similar effects for both RNM intercept and slope for NW, OW, MD, and BF (with one exception on SSC1 that was associated independently with RNM intercept).

A total of 20 genes, including 16 protein-encoding and four lncRNA, were found within these candidate genomic regions. There was no significant GO biological process or KEGG pathway according to the functional enrichment analyses. However, five candidate genes were significantly involved in one or more biological processes and some of these genes have positive biological implications (see Additional file 2 Figure S6), such as the GO term of “negative regulation of retinoic acid receptor signaling pathway” for BF. The two genomic regions on SSC10 and SSCX of NW are linked to multiple reported QTLs of health-related traits in pigs, including melanoma susceptibility, osteochondrosis score, and immunity response (see Additional file 1 Table S6). The three genomic regions on SSC1, SSC13, and SSC14 found for OW are supported by a series of known QTLs for growth traits in pigs. Additionally, we found very strong evidence for MD and BF, because these significant genomic regions were linked to several known QTLs for muscle and carcass fatness traits.
